# A novel pathway-based distance score enhances assessment of disease heterogeneity in gene expression

**DOI:** 10.1186/s12859-017-1727-4

**Published:** 2017-06-20

**Authors:** Xiting Yan, Anqi Liang, Jose Gomez, Lauren Cohn, Hongyu Zhao, Geoffrey L. Chupp

**Affiliations:** 1 Center for Pulmonary Personalized Medicine, Section of Pulmonary, Critical Care, and Sleep Medicine, Department of Internal Medicine, Yale School of Medicine, New Haven, CT 06520 USA; 2 Department of Biostatistics, Yale School of Public Health, New Haven, CT 06520 USA; 3 Department of Genetics, Yale School of Medicine, New Haven, CT 06520 USA; 4 Computational Biology and Bioinformatics Program, Yale School of Medicine, New Haven, CT 06520 USA

**Keywords:** Data integration, Unsupervised clustering, Disease heterogeneity, Pathway-based distance

## Abstract

**Background:**

Distance based unsupervised clustering of gene expression data is commonly used to identify heterogeneity in biologic samples. However, high noise levels in gene expression data and relatively high correlation between genes are often encountered, so traditional distances such as Euclidean distance may not be effective at discriminating the biological differences between samples. An alternative method to examine disease phenotypes is to use pre-defined biological pathways. These pathways have been shown to be perturbed in different ways in different subjects who have similar clinical features. We hypothesize that differences in the expressions of genes in a given pathway are more predictive of differences in biological differences compared to standard approaches and if integrated into clustering analysis will enhance the robustness and accuracy of the clustering method. To examine this hypothesis, we developed a novel computational method to assess the biological differences between samples using gene expression data by assuming that ontologically defined biological pathways in biologically similar samples have similar behavior.

**Results:**

Pre-defined biological pathways were downloaded and genes in each pathway were used to cluster samples using the Gaussian mixture model. The clustering results across different pathways were then summarized to calculate the pathway-based distance score between samples. This method was applied to both simulated and real data sets and compared to the traditional Euclidean distance and another pathway-based clustering method, Pathifier. The results show that the pathway-based distance score performs significantly better than the Euclidean distance, especially when the heterogeneity is low and genes in the same pathways are correlated. Compared to Pathifier, we demonstrated that our approach achieves higher accuracy and robustness for small pathways. When the pathway size is large, by downsampling the pathways into smaller pathways, our approach was able to achieve comparable performance.

**Conclusions:**

We have developed a novel distance score that represents the biological differences between samples using gene expression data and pre-defined biological pathway information. Application of this distance score results in more accurate, robust, and biologically meaningful clustering results in both simulated data and real data when compared to traditional methods. It also has comparable or better performance compared to Pathifier.

**Electronic supplementary material:**

The online version of this article (doi:10.1186/s12859-017-1727-4) contains supplementary material, which is available to authorized users.

## Background

The pathogenetic causes of many diseases have been known to be heterogeneous, including different types of cancers and chronic inflammatory diseases of the lung and other organs [1–3]. This heterogeneity contributes to differences in clinical manifestations of disease and response to therapeutic intervention. This suggests that precisely defining pathogenically relevant subtypes or “endotypes” of disease will improve the predicted response to a given therapy, especially in complex chronic diseases. Global gene expression analysis has been successfully applied to identify the molecular subtypes or endotypes that are associated with the clinical heterogeneity [4–7] and promises to pave the way to identify both the biology of disease pathogenesis and endotypes of disease that can be treated more precisely.

Distance based unsupervised clustering methods have been among the most popular approaches to identify biological heterogeneity from gene expression data. Usually, the original gene expression data is filtered based on the variance of the expression levels across the samples being analyzed. Many studies followed this analysis framework and successfully identified clinically or biologically meaningful disease subtypes [6, 8–10]. However, these approaches have major limitations which may render them ineffective under certain circumstances. First, most of the reported studies select genes based on the variance of their expression levels. However, since multiple studies have shown that disease-associated and causing genes do not necessarily have high gene expression levels and thus do not demonstrate a large variation, selecting genes based on their variance may result in a poor discrimination of biologically relevant disease subtypes [11, 12]. Second, the Euclidean distance assigns equal weight to all genes included in the analysis. It is known that different genes can be perturbed to a different extent by the same stimulus, so assigning an equal weight is biologically inaccurate. Furthermore, perturbations in genes that interact with many other genes tend to have a larger biologic effect on the disease phenotype [13–15]. Therefore, different genes should not be treated equally but should be weighted to reflect the strength of any given association with the clinical phenotype. Third, genes that function together, including those in the same biological pathway, tend to have strong correlation in their expression levels. This correlation is not accounted for by the Euclidean distance. Lastly, using a measure of multiple genes in a pathway will limit the noise that is inherent in gene expression data.

To address these issues, we developed a novel distance score that assesses the biological differences between samples by integrating pathway information based on the assumption that biologically similar samples tend to have similar expression patterns of biological pathways. Pre-defined biological pathways are selected to assess the biological difference between samples. We use genes from each pathway to cluster the samples based on a multivariate Gaussian mixture model. Then, the clustering results across all the pathways are summarized into a distance score that is small when most of the pathways assign two given samples into the same cluster. This distance score has three advantages over the traditional Euclidean distance. First, it takes advantage of the pre-defined biological pathways which include genes that are more likely to be disease or phenotype associated. This results in less background noise for clustering. Second, clustering results using pathways are more robust than using single genes due to the high noise levels in the gene expression data. Third, the multivariate Gaussian mixture model accounts for the correlation between genes from the same pathways which makes the clustering results more accurate.

The incorporation of biological knowledge into clustering methods has been proposed before. Several previous studies have recognized the benefit of using the ontological information to identify the disease heterogeneity from genetic mutations [16–19], protein changes [20, 21], transcriptomic data [22–30] and a combination of genomic and transcriptomic data [31]. Multiple pathway-based clustering methods have been developed by these studies. The Pathifier [22] performs a principal component analysis for each pathway to project the samples onto a subspace formed by the top components explaining >10% of the variation. In the subspace, a principal curve is formed and all the samples are projected onto this curve. The distance of each sample from a consensus or control sample on this curve will be considered as the pathway activity score of the given pathway in the given sample. PathVar [29] computes an expression variance matrix for each pathway using three metrics that measure the variability of the genes inside the pathway. This expression variance matrix was then used to cluster samples to identify sample groups with similar expression variance across multiple pathways. The study by Verhaegh et al. [23] predicts signaling pathway activity based on knowledge-based Bayesian network models, which interpret the expression patterns of the manually picked target genes of pathways as the functional output of the activity of the pathways. Zhao et al. [19] clustered samples using a voting mechanism which is very similar to our proposed approach, but with a major difference in how each pathway clusters the samples. The study by Lottaz et al. [28] incorporated the Gene Ontology (GO) hierarchy information to cluster samples with different clinical phenotypes based on microarray gene expression data. However, due to the lack of a hierarchical structure of genes involved in the same biological pathways, this method cannot be applied if the prior knowledge comes from the biological pathways available from many online databases. These developed methods have been successful in identifying novel subtypes of diseases, especially in cancers. However, when applied to transcriptomic data from chronic diseases, these developed methods have certain limitations. For example, both Pathifier and PathVar rely on the assumption that genes that are strongly associated with the underlying disease pathogenesis have much higher variation than other genes, which might not be true for chronic diseases. Chronic diseases are known to have smaller changes in both genome and transcriptome compared to cancers, which will make the top components explain a smaller percentage of variation and also likely cause the top components to have less association with the underlying disease pathogenesis. The Bayesian network model used by Verhaegh et al. requires and heavily relies on the knowledge on the direct target genes of pathways. Currently, there is no accurate source for this information. Besides, the target genes of pathways might vary between individuals, tissues, and diseases. Zhao et al. use hierarchical clustering to cluster samples using each pathway, which is not a very accurate and robust clustering approach. The pathway-based distance score that we developed enhances for heterogeneity associated gene signatures and reduces the noise level by summarizing the clustering results across multiple Gaussian mixture models that integrate prior pathway information.

We applied the proposed method to both simulated data and real data and compared it to the traditional Euclidean distance with and without gene filtering as well as Pathifier. The results from simulated data show that our method performs better than the traditional Euclidean distance coupled with K-means clustering or hierarchical clustering, especially when the percentage of genes that are perturbed in the pathway is high, the perturbed genes have large changes in their expression levels and there is strong correlation between the expression levels of genes from the same pathway. Compared to Pathifier, our method shows higher clustering accuracy and better robustness to background noise for small pathways. By adding an extra step of downsampling the pathways, our approach achieves comparable performance to Pathifier for bigger pathways. Application to a real dataset in asthma patients identified 3 subgroups which are associated with important clinical features of asthma. These associated clinical features have been further validated in an independent cohort demonstrating the power of the proposed method. In contrast, when traditional unsupervised clustering methods and Pathifier were applied, the identified clusters were associated with less clinical features and had weaker association strengths. Application to another real dataset from non-small cell lung cancer patients shows comparable performances of all methods, indicating that the perturbations in the transcriptome of cancer patients are so high that all methods will achieve the same performance. In summary, the application of our method to both simulated data and real data showed that the proposed method has a better performance in identifying disease heterogeneity than the Euclidean distance with or without gene filter. It also has equal or better performance than Pathifier and it is more likely to perform better in chronic diseases with relatively weaker signals.

## Methods

### Pathway-based distance score

Let *G* = (*g*_*ij*_)_*M* × *N*_ be a matrix with *M* rows and *N* columns, in which rows and columns correspond to genes and subjects respectively, and *g*_*ij*_ is the expression level of gene *G*_*i*_ in subject *S*_*j*_. The pre-defined biological pathways, denoted as *P* = {*P*_*k*_ : *k* = 1, 2,  ⋯ , *K*}, provide the definition of pathways, where ${P}_k=\left\{{G}_{i_1^k},{G}_{i_2^k},\cdots, {G}_{i_{m_k}^k}\right\}$ is the set of genes in pathway *P*_*k*_. To calculate the pathway-based distance score between samples, we first cluster all the samples using the expression levels of the member genes from each pathway separately. The multivariate Gaussian Mixture Model is used for the clustering, which selects the number of clusters based on the Bayesian Inference Criterion (BIC). Suppose that pathway *P*_*k*_ suggests that there are *m*_*k*_ clusters and the clustering results are denoted as ${C}_k=\left({c}_1^k,{c}_2^k,\cdots, {c}_N^k\right)$, in which $1{\le c}_j^k\le {m}_k$ and ${c}_j^k$ is an integer representing the cluster assignment of the subject *S*_*j*_ based on member genes from pathway *P*_*k*_. The pathway-based distance score between subjects *j*_1_ and *j*_2_ is then defined as

$$ d\left({j}_1,{j}_2\right)=\frac{\#\left\{ k:{c}_{j_1}^k\ne {c}_{j_2}^k,{m}_k>1\right\}}{\#\left\{ k:{m}_k>1\right\}}, $$

where #{·} is the size of the set {·}. We exclude the pathways that only identify one cluster, and the distance score is the proportion of these filtered pathways that assign the two subjects into different clusters. Since this score is not a true distance, we treat this scoring matrix as a new data matrix in which each column is one subject. Results, when this scoring matrix is treated as a distance matrix for hierarchical clustering method, can be found in Additional file 1: Figure S1, which shows a significant improvement in the connectivity plot by considering the scoring matrix as a new data matrix instead of a distance matrix. The final distance between two subjects will be calculated as the Euclidean distance between the two corresponding columns from the scoring matrix.

### Downsampling pathways

When there are *p* genes in one pathway, the Gaussian mixture model with one component will need to estimate roughly (*p* ^2^ + 3*p*)/2 parameters with (*p* ^2^ + *p*)/2 of them from the covariance matrix and the other *p* of them from the mean. So, for a small sample size (~100), it is very easy for the model to have much larger number of parameters to estimate than the number of observations, which can also be seen from Additional file 1: Figure S9. Under this circumstance, to improve the performance of the pathway-based distance score, we downsample the pathways into smaller pathways. For the data simulated by the high dimension simulation model, we randomly sample 100 subsets of 10 genes from each pathway and apply Gaussian mixture model to cluster the samples using each of these 100 subsets of genes. Then the distance between two samples is calculated as the proportion of subsets of genes that cluster the two samples into the different clusters. This new distance matrix will then be used to cluster the samples by finding the optimal number of clusters, first using connectivity criterion and then applying K-means with K being the identified optimal K. In this way, each pathway will provide one clustering result and the final distance score is calculated in the way described in section 2.1. The optimal choice of the number of random sampling depends on the pathway size and the optimal choice of the number of genes to be sampled for each random sampling depends on the sample size. When sample size is bigger, the Gaussian mixture model will be able to accurately estimate more parameters so we can choose a larger number of genes to sample for each subset. And when the pathways have more member genes, we will need to increase the number of random sampling so that there will be enough number of subsets that contain a decent number of genes with signal. In this article, we simulated 120 subjects and the size of the KEGG pathways ranges from 6 to over 360. We chose the number of genes to sample to be 10 based on the simulation results and, for each pathway, we did the random sampling 100 times (for which we do not have any evidence and there might be ways to improve this setting).

### Distance by Pathifier

To calculate the distance between samples using Pathifier, we apply Pathifier to the expression data of genes from each pathway, which provides a pathway activity score for the given pathway in each of the subjects. The distance between any two subjects is then calculated as the Euclidean distance between their pathway activity scores from all pathways.

### Data simulation

To demonstrate the performance of the method, we simulated multiple gene expression data sets using different parameter settings. We assume a total of 22,148 genes were measured, which is the same as the total number of genes measured on the Affymetrix HuGene 1.0 ST chip used in the real data. These genes were assigned to either a set of artificially defined pathways or the 186 KEGG pathways by MsigDB [32]. Among the 22,148 genes, 4841 genes were assigned to at least one KEGG pathway. We assume that there are 120 samples evenly divided into 3 groups. In each group, a subset of pathways is randomly selected to be associated with the grouping. Within each of these selected pathways, a subset of its member genes is randomly chosen to be differentially expressed between the 3 groups.

Suppose the subjects are denoted as (*S*_1_, *S*_2_,  ⋯ , *S*_120_) and the cluster that subject *S*_*i*_ belongs to is *C*_*i*_. We assume that

$$ {C}_i=\left\{\begin{array}{c}1,\kern0.5em  i f\  i=1,2,\cdots, 40\\ {}2,\kern0.5em  i f\  i=41,42,\cdots, 80\\ {}3, if\  i=81,82,\cdots, 120\end{array}\right. $$,

which means that the first 40 samples belong to group 1, the second 40 samples belong to group 2 and the last 40 samples form group 3. To simulate the gene expression profile, we first randomly choose a given percentage (*p*_*W*_) of the pre-defined pathways to be associated with the grouping. For example, if *P*_*W*_ = 0.2, we randomly choose 37 pathways. Then for each chosen pathway *P*_*k*_, we randomly select a given percentage (*p*_*G*_) of its member genes to be differentially expressed across the 3 groups. Let *g*_*ij*_ be the expression level of gene *j* in subject *S*_*i*_, Ω_k_ be the set of genes from pathway *P*_*k*_ that was chosen to be differentially expressed, and ${G}_{i{\Omega}_{\mathrm{k}}}$ be the vector of expression levels of genes in Ω_k_ from subject *S*_*i*_. Then the gene expression levels of all genes in pathway *P*_*k*_ will have the following distribution:

$$ \left(\genfrac{}{}{0pt}{}{G_{i\ {\varOmega}_k}}{G_{i\overset{-}{\varOmega_k}}}\right)\sim Gaussian\left(\left(\genfrac{}{}{0pt}{}{\mu_{C_i}}{0}\right),\varSigma =\left(\begin{array}{cc}{\varSigma}_0& \rho \varPi \\ {}\rho \varPi & {\varSigma}_1\end{array}\right)\right), $$

in which

$$ {\mu}_{C_i}=\left\{\begin{array}{c}-\delta, if\ {C}_i=1\\ {}0, if\ {C}_i=2\\ {}\delta, if\ {C}_i=3\end{array}\right. $$, $$ {\Sigma}_0=\left[\begin{array}{ccc}{\sigma}^2\ & \cdots & \rho \\ {}\vdots & \ddots & \vdots \\ {}\rho & \cdots & {\sigma}^2\end{array}\right] $$, *σ* ^2^ = 1 + 2*δ* ^2^/3, $$ {\varSigma}_1=\left[\begin{array}{ccc}{B\sigma}^2\ & \cdots & \rho \\ {}\vdots & \ddots & \vdots \\ {}\rho & \cdots & B{\sigma}^2\end{array}\right] $$ and $$ \Pi =\left[\begin{array}{ccc}1\ & \cdots & 1\\ {}\vdots & \ddots & \vdots \\ {}1& \cdots & 1\end{array}\right] $$.

By this simulation model, the gene expression profile of subject *S*_*i*_ is assumed to follow a multivariate normal distribution with mean ${\mu}_{C_i}$ and covariance matrix Σ, which indicates that subjects from the same group have the same gene expression profile distribution. We set the marginal standard deviation of the chosen genes to be 1 + 2*δ* ^2^/3 so that, for each group, we can simulate the gene expression levels of each individual from a multivariate Gaussian distribution with marginal variance of 1 for all the chosen genes. The final simulated data can be generated by simply merging the simulated expression levels for all individuals together. The simulation model also assumes that the expression levels of genes from pathways that were not chosen to be associated with the grouping have the same multivariate Gaussian distribution for all individuals, with a mean of 0 for all genes, regardless of what cluster the subject belongs to. The marginal variance of the non-chosen genes is set to be *Bσ* ^2^(*B* = 1,1.5,2) so that we can introduce different levels of noise in the simulated data to show and compare the robustness of the methods. For each given setting of *p*_*W*_, *p*_*G*_, *δ* , *B* and *ρ*, we simulated 100 data sets and applied different approaches to compare their performance.

To better understand the performance of our approach, we simulated the data in two different ways: low dimension and high dimension. For the low dimension simulation, we artificially generated a set of 186 pre-defined pathways by pooling all genes annotated in the 186 KEGG pathways and sampling from them without replacement to form equally sized and non-overlapping 186 pathways. For the high dimension simulation, we directly used the 186 KEGG pathways from MsigDB.

### Clustering methods performance evaluation

We evaluate the performance of different clustering approaches for accuracy and robustness. Accuracy is evaluated in two ways. First, we assess the ability of each approach to identify the correct number of clusters. For each approach, we calculate the internal clustering criterion (connectivity and Dunn Index [33]) for different numbers of clusters. The connectivity criterion is defined to measure the difference between the given clustering results and the neighborhood structure of all the samples. Let *C* = {*c*_1_, *c*_2_,  ⋯ , *c*_*K*_} be a given clustering result of *N* samples that divides the samples into *K* clusters. Define *nn*_*i*(*j*)_ as the *j*-th nearest neighbor of sample *i* based on one of the four different types of distances and let ${\delta}_{i, n{n}_{i(j)}}$ be zero if sample *i* and *j* are in the same cluster and 1/*j* otherwise. Then the connectivity of the clustering result *C* using a given distance measure is defined as $connectivity(C)=\sum_{i=1}^N\sum_j^L{x}_{i, n{n}_{i(j)}}$, where *L* is a parameter giving the number of nearest neighbors to include for each sample. So the connectivity criterion is large when the neighbors of the samples are assigned to different clusters, indicating a low quality of the given clustering results. The nearer these misclassified neighbors are to the samples, the larger the connectivity criterion is. The value of the connectivity criterion varies between 0 and ∞ and should be minimized. The optimal number of clusters is chosen to be the value that optimizes the internal clustering criterion. Among all the 100 simulated data sets with the same parameter setting, we count the number of data sets that identify 3 as the optimal number of clusters and use this as the first measure of performance. Second, we evaluate the ability of different approaches in finding the correct clustering results. For each simulation, we apply K-means and hierarchical clustering to the distance matrix from each approach by setting the required number of clusters to be the optimal number of clusters chosen by the corresponding approach. The clustering results are compared to the true clustering results by calculating the purity criterion [33] which measures the differences between a given clustering result and the true grouping. For robustness, we vary the value of *B* to introduce different levels of noise in the simulation model and compare the accuracy of different methods across these different noise levels to investigate how robust the methods are to background noise.

## Results

To demonstrate the performance of our approach, we compared it to three other approaches including Pathifier, the Euclidean distance based on all genes and genes included in the simulated pre-defined pathways or the KEGG pathways, respectively. The comparison was done using both simulated data and two real datasets.

### Simulated data

For the simulated data, we set the percentage of perturbed pathways (*p*_*W*_) to be 20% and vary the percentage of perturbed genes per pathway (*p*_*G*_) to be 20%, 40%, 60% and 80%. The correlation coefficient between perturbed genes from the same pathway (*ρ*) varies from 0 to 0.9, and the differences in the expression levels between different groups (*δ*) vary from 0.5 to 1.5. The higher *δ* is, the easier it should be for the methods to identify the correct clustering results. But for *ρ*, this may not be true. We applied both K-means clustering and hierarchical clustering to the simulated data using the distance matrix calculated in four ways: Euclidean distance using all genes, Euclidean distance using genes from all 186 KEGG pathways, Euclidean distance of the pathway activity scores calculated by Pathifier, and our pathway-based distance score. The Euclidean distance using all genes represents the situations when no prior information is integrated, while the Euclidean distance using the KEGG genes and the Pathifier represents the situations when the prior pathway information is used to filter genes only. We show the comparison of the pathway-based distance score to these methods to demonstrate the benefit of both filtering genes correctly and calculating the distance based on sets of functionally related genes or pathways instead of individual genes. The comparison to Pathifier will show the benefit of different approaches to integrate the pathway information and their corresponding favorable situations.

#### Low dimension independent model

**Fig. 1:**
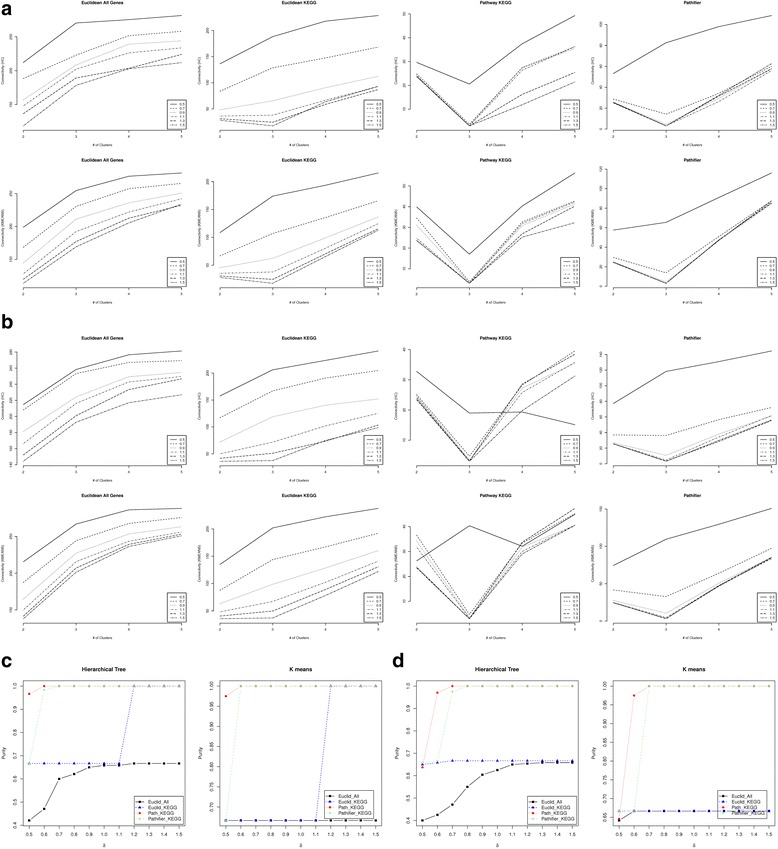
Performance comparison when *ρ*=0 and *B*=1 for low dimension simulation. The median connectivity when *p*_*G*_ = 0.8 (panel **a**) and *p*_*G*_ = 0.6 (panel **b**) for different numbers of clusters using four distances: Euclidean distance using all genes (Euclidean All Genes), Euclidean distance using KEGG covered genes only (Euclidean KEGG), KEGG pathway-based distance score (Pathway KEGG) and the Euclidean distance of the pathway activity scores calculated by Pathifier (Pathifier). Both the hierarchical tree clustering (HC) and the K-means (KMEANS) were used to calculate the connectivity criteria. Different lines in each panel represent the connectivity across the different number of clusters for each given value of δ = 0.5,0.7,0.9,1.1,1.3,1.5. The median purity criterion of the clustering results on the 100 simulated data sets when hierarchical clustering and K-means are applied to the four distances when *p*_*G*_ = 0.8 (panel **c**) and *p*_*G*_ = 0.6 (panel **d**). The number of clusters was set to be the optimal number of clusters identified based on the connectivity criteria using the corresponding calculated distance

We first examined the results of the low dimension simulation with 10 member genes per pathway (S = 10) and no correlation between genes, i.e. ρ = 0. When B = 1 , p_G_ = 0.6, and δ varies from 0.5 to 1.5, we calculated the median connectivity (Fig. 1, standard deviation shown in Additional file 1: Figure S2) and Dunn Index (Additional file 1: Figure S3) across the 100 simulated data sets of all the four types of distances for given numbers (2,3,4,5) of clusters. The same results for p_G_ = 0.4 can be found in Additional file 1: Figure S4. As shown in Fig. 1a and b, across different numbers of clusters, both the pathway-based distance score and Pathifier achieve the minimum connectivity criterion at the true number of clusters (k = 3) consistently, except when *p*_*G*_ = 0.6 and *δ* is smaller than 0.7. Euclidean distance using KEGG genes starts to identify the right number of clusters when *δ* becomes higher than 1.3. The median connectivity criterion by the Euclidean distance using all genes never identifies the right number of clusters for any *δ*, no matter what *p*_*G*_ is. Between our approach and Pathifier, when *δ* = 0.5 and *p*_*G*_ = 0.8, indicating that the differences between different groups are very small but a high percentage of genes are differentially expressed, our approach still achieves the minimum connectivity for 3 but Pathifier does not. Next, for each distance, we set the number of wanted clusters to be the identified optimal number of clusters based on the connectivity criterion and apply both hierarchical clustering and K-means clustering with the distance to cluster the samples. The clustering results were then compared to the true classes of all the 120 samples to calculate the purity criterion, and are shown in Fig. 1c and d. The comparison shows that both our approach and Pathifier outperform the other two distances, especially when *δ* is small. When *δ* becomes higher than 1.3, the Euclidean distance using the KEGG pathways annotated genes becomes comparable. However, the Euclidean distance using all genes always has the smallest purity for the whole range of *δ*, indicating the importance of filtering genes in the right way. Then, between our approach and Pathifier, they achieve the same high purity level when *δ* > 0.7. But when *p*_*G*_ = 0.8, Pathifier has lower purity level mainly because of its failure to identify the true number of clusters. When *p*_*G*_ decreases to 0.6, both our approach and Pathifier fail to identify the true number of clusters when *δ* = 0.5. But Pathifier has slightly higher purity than our approach, because of the fact that the distance by Pathifier is continuous. Thus, even when there are no clusters, the distance can still provide certain but low information about the differences between samples. While our approach tends to assign the distance score to be all 0 for all pairs of samples when the differences between groups are extremely small and the total number of pathways is low, our approach will have no pathway that identifies more than one clusters causing the distance scores to be all 0 for all pairs of samples. When *δ* increases to 0.6, both our approach and Pathifier will identify the correct number of clusters, but our approach has higher purity than Pathifier. To summarize, for low dimensional pathways, our approach and Pathifier have the same performance when there are decent differences between different groups. When the group differences decrease, as long as our approach is still able to identify the correct number of clusters, its clustering results have higher purity than Pathifier. Of course, due to the way the pathway-based distance score is defined, when the group difference is so low that no method can identify the correct number of clusters, Pathifier will have higher purity than our approach. We expect our approach to perform better when the number of pathways is higher, since it will increase the chance of having pathways identifying more than one cluster.

**Fig. 2:**
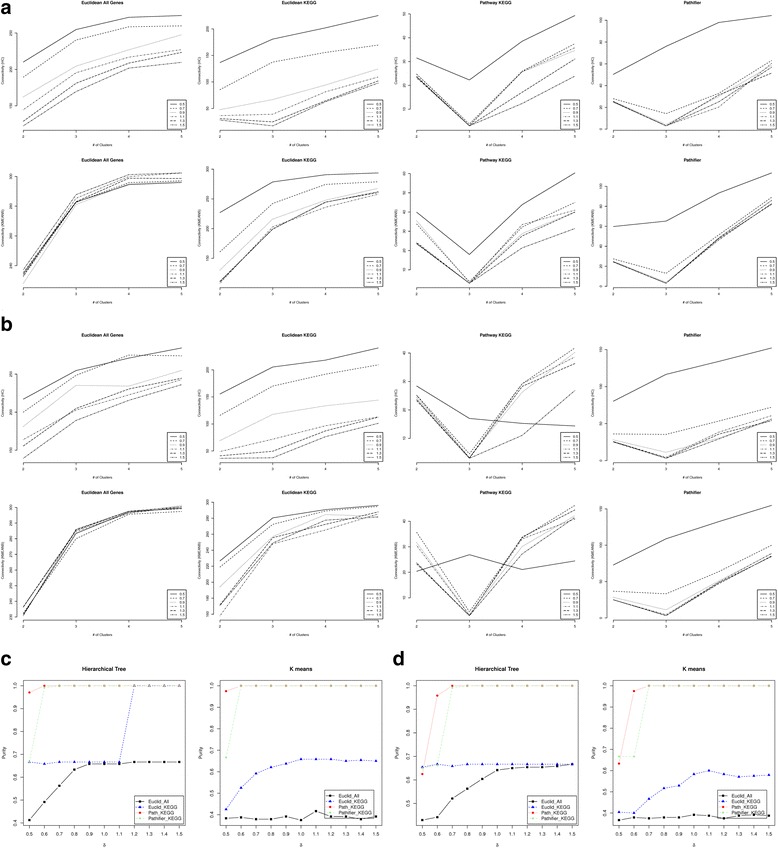
Performance comparison when *ρ*=0 and *B*=3 for low dimension simulation. The median connectivity when *p*_*G*_ = 0.8 (panel **a**) and *p*_*G*_ = 0.6 (panel **b**) for different numbers of clusters using four distances: Euclidean distance using all genes (Euclidean All Genes), Euclidean distance using KEGG covered genes only (Euclidean KEGG), KEGG pathway-based distance score (Pathway KEGG) and the Euclidean distance of the pathway activity scores calculated by Pathifier (Pathifier). Both the hierarchical tree clustering (HC) and the K-means (KMEANS) were used to calculate the connectivity criteria. Different lines in each panel represent the connectivity across the different number of clusters for each given value of δ = 0.5,0.7,0.9,1.1,1.3,1.5. The median purity criterion of the clustering results on the 100 simulated data sets when hierarchical clustering and K-means are applied to the four distances when *p*_*G*_ = 0.8 (panel **c**) and *p*_*G*_ = 0.6 (panel **d**). The number of clusters were set to be the optimal number of clusters identified based on the connectivity criteria using the corresponding calculated distance

To compare the robustness of the methods, we set *B*=3 to introduce a much higher level of background noise in the data. The accuracy of the four methods for *B*=3 can be found in Fig. 2 (standard deviation of the connectivity in Additional file 1: Figure S5) and the Dunn Index can be found in Additional file 1: Figure S6. Corresponding results for *p*_*G*_ = 0.4 can be found in Additional file 1: Figure S7. When comparing Fig. 2 to Fig. 1, we found that when *B* increases from 1 to 3, Pathifier fails to identify the correct number of clusters for *δ* = 0.5, while our approach is still able to find 3 as the optimal K. When comparing Additional file 1: Figure S7 to Figure S4, the difference is not as significant. This indicates that the pathway-based distance score is more robust to background noise than Pathifier, especially when there are many genes in the pathways associated with the grouping.

#### High dimension independent model

**Fig. 3:**
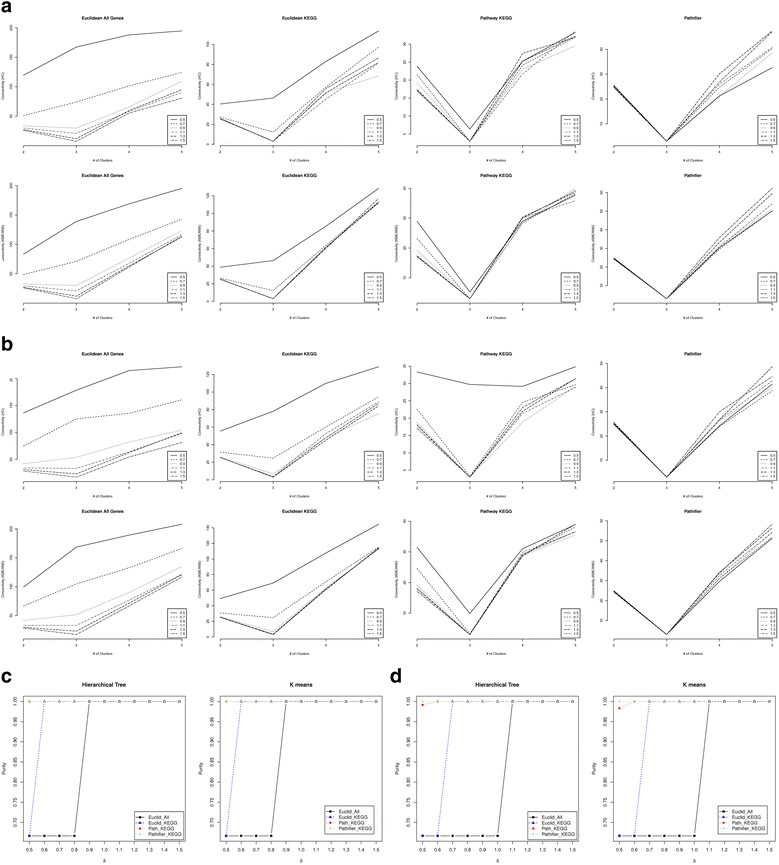
Performance comparison when *ρ*=0 and *B*=1 for high dimension simulation. The median connectivity when *p*_*G*_ = 0.8 (panel a) and *p*_*G*_ = 0.6 (panel **b**) for different numbers of clusters using four distances: Euclidean distance using all genes (Euclidean All Genes), Euclidean distance using KEGG covered genes only (Euclidean KEGG), KEGG pathway-based distance score (Pathway KEGG) and the Euclidean distance of the pathway activity scores calculated by Pathifier (Pathifier_KEGG). Both the hierarchical tree clustering (HC) and the K-means (KMEANS) were used to calculate the connectivity criteria. Different lines in each panel represent the connectivity across the different number of clusters for each given value of δ = 0.5,0.7,0.9,1.1,1.3,1.5. The median purity criterion of the clustering results on the 100 simulated data sets when hierarchical clustering and K-means are applied to the four distances when *p*_*G*_ = 0.8 (panel **c**) and *p*_*G*_ = 0.6 (panel **d**). The number of clusters was set to be the optimal number of clusters identified based on the connectivity criteria using the corresponding calculated distance

**Fig. 4:**
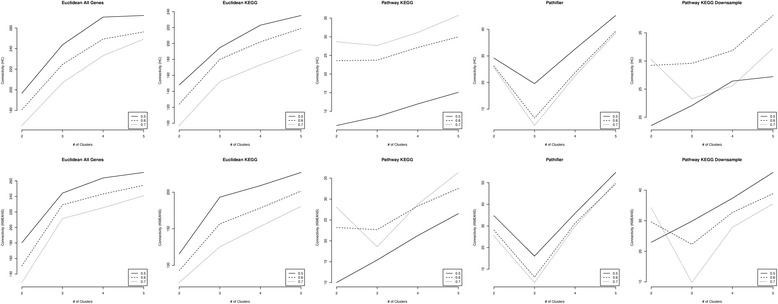
Median connectivity criteria when pathways are down sampled in the high dimension simulation model. The connectivity of the Euclidean distance using all genes (Euclidean All Genes), using KEGG genes (Euclidean KEGG), pathway-based distance score using KEGG pathways (Pathway KEGG), Pathifier distance using KEGG pathways (Pathifier) and the pathway-based distance score with pathway downsampling (Pathway KEGG Downsample) are shown for each given *δ*=0.5, 0.6 and 0.7. In this simulation, *B*=1 and *p*_*G*_=0.2

**Fig. 5:**
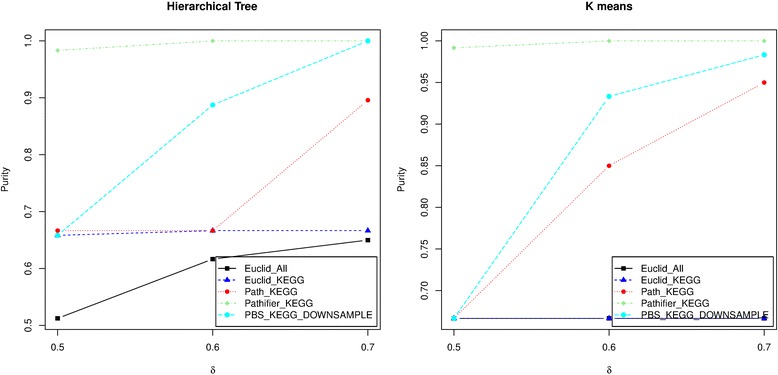
Median purity when pathways are down sampled in the high dimension simulation model. Median purity of the Euclidean distance using all genes (Euclidean_All), using KEGG genes (Euclidean_KEGG), pathway-based distance score using KEGG pathways (Pathway_KEGG), Pathifier distance using KEGG pathways (Pathifier_KEGG) and the pathway-based distance score with pathway down sampling (PBS KEGG Downsample) are shown for each given *δ*=0.5,0.6 and 0.7. In this simulation, *B*=1 and *p*_*G*_=0.2

For the high dimension simulation model, we set *p*_*G*_=0.2, 0.4, 0.6 and 0.8 and examined the results when there is no correlation between genes, i.e. *ρ* = 0. When *δ* varies from 0.5 to 1.5, the median connectivity criterion across the 100 simulated data sets of the four types of distances for a given number of clusters (2,3,4,5) is shown in Fig. 3. The results show that, across different numbers of clusters, the pathway-based distance score achieves the minimum connectivity criterion at the true number of clusters (k = 3) consistently, except when *δ* = 0.5 and hierarchical clustering is used to calculate the connectivity criterion. Pathifier, however, always identifies the correct number of clusters no matter what *δ* is. Again, Euclidean distance using KEGG genes starts to identify the right number of clusters when *δ* becomes higher than 0.5. The median connectivity criterion by the Euclidean distance using all genes starts to identify the right number of clusters when *δ* > 0.9. The actual percentage of simulated datasets for which the four types of distances identify the correct number of clusters (k = 3) based on the connectivity criterion is shown in Table 1. The pathway-based distance score and Pathifier always achieve the highest percentage of the correctly identified number of clusters. As the differences between different clusters (*δ*) increases, the Euclidean distance using KEGG genes becomes better and comparable to the pathway-based distance score and Pathifier in terms of its ability to find the right number of clusters. In addition, the purity comparison in Fig. 3c and d show that both the pathway-based distance score and the Pathifier outperform the other two distances, especially when *δ* is small, indicating the benefit of integrating pathway information. When *δ* becomes higher than 0.6, the Euclidean distance using the KEGG pathways annotated genes becomes comparable to the pathway-based distance score. And, the Euclidean distance using all genes always becomes comparable to the other methods when *δ* > 0.9, indicating the importance of filtering genes in the right way. Between Pathifier and the pathway-based distance score, when *p*_*G*_=0.2, Pathifier has much higher purity than the pathway-based distance score especially for *δ* < 0.9 (Additional file 1: Figure S8). A closer investigation of the results revealed that the mclust R package that we used for the Gaussian mixture model clustering becomes less efficient when the size of the pathway increases (Additional file 1: Figure S9). To improve this, we down sampled all the pathways down to 100 subsets of 10 genes for *B*=1, *δ*=0.5, 0.6 and 0.7, and *p*_*G*_=0.2 and the results are shown in Figs. 4 and 5. The figures show that although the downsampling strategy does not improve the performance of the pathway-based distance score in identifying the correct number of clusters, the corresponding purity of the clustering results does significantly improve. With this very rough downsampling strategy, the pathway-based distance score achieves comparable performance when *δ* > 0.6 compared to *δ* > 0.9 without this downsampling step. Again, we chose the number of genes to sample to be 10 since the simulation results with 10 genes per pathway showed outer performance of our approach. But, we did the random sampling 100 times for each pathway without any evidence. We believe that finer tuning on the number of random samplings can further improve the performance.

**Table 1:** The accuracy rate of identifying the true number of clusters when *ρ* =0, *B* = 1 and *p*_*G*_ = 0.2

*δ*		0.5	0.6	0.7	0.8	0.9	1.0	1.1	1.2	1.3	1.4	1.5
HC	Euclid All	13%	13%	10%	8%	7%	7%	2%	6%	1%	10%	7%
	Euclid KEGG	6%	8%	2%	3%	4%	2%	3%	19%	35%	55%	72%
	Path KEGG	22%	37%	34%	38%	45%	61%	75%	89%	98%	99%	100%
Kmeans	Euclid All	0%	0%	0%	0%	0%	0%	0%	0%	0%	0%	0%
	Euclid KEGG	0%	0%	0%	0%	0%	0%	3%	19%	39%	54%	77%
	Path KEGG	19%	50%	77%	92%	97%	97%	100%	100%	100%	99%	100%

When there is no correlation between genes, for different values of δ, the percentage of simulated data sets for which the given distances identify 3 as the optimal number of clusters based on the connectivity criteria is shown. Both hierarchical tree (HC) and K-means (Kmeans) were used as clustering method

#### High dimension dependent model

**Fig. 6:**
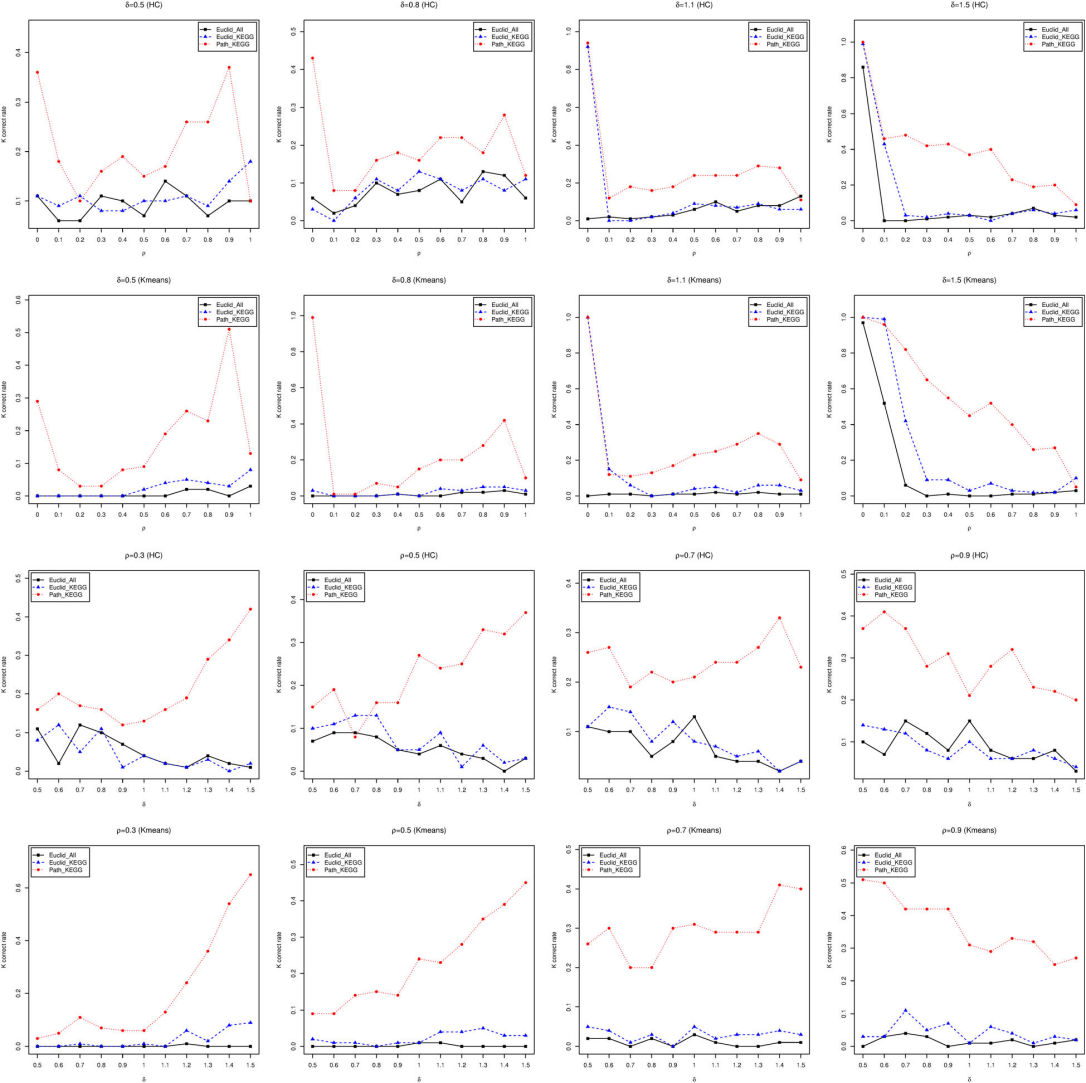
The accuracy of identifying the true number of clusters when genes are dependent. The percentage among the 100 simulated data sets that identified 3 as the optimal number of clusters based on the connectivity criterion calculated from the three distances. The top two rows of panels show this percentage versus ρ for given δ=0.5, 0.8, 1.1 and 1.5. The bottom two rows of panels show the percentage versus δ for given ρ=0.3, 0.5, 0.7 and 0.9. Both hierarchical clustering (HC) and K-means clustering (Kmeans) were used to calculate the connectivity criteria

**Fig. 7:**
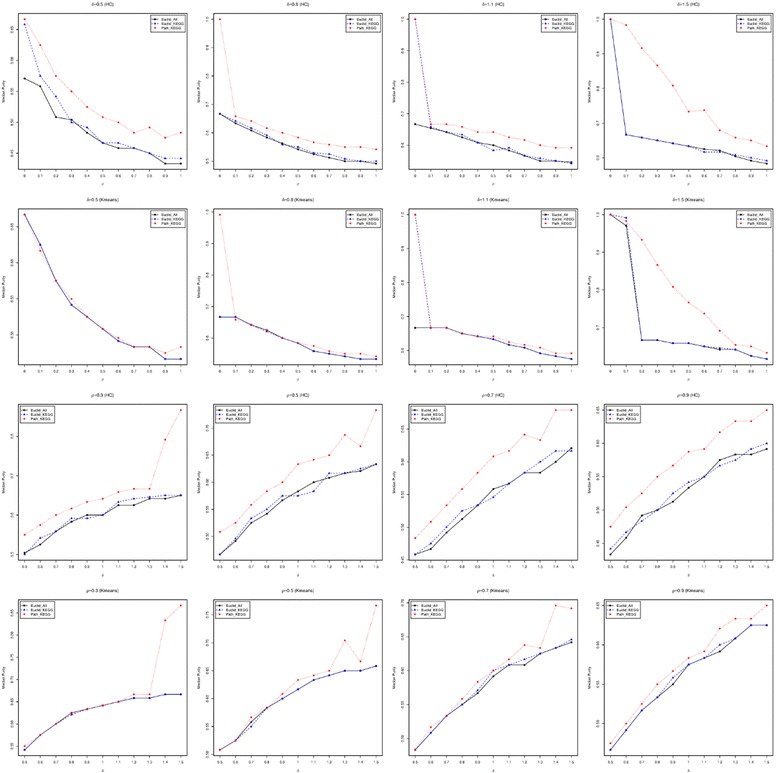
The accuracy of the clustering results when genes are dependent. The median purity criterion of the clustering results by hierarchical clustering (HC) and K-means clustering (Kmeans) using the three distances. The top two rows of panels describe the median purity criteria versus *ρ* for each given *δ*=0.5, 0.8, 1.1 and 1.5. The bottom two rows of panels describe the median purity criteria versus *δ* for each given *ρ* = 0.3 , 0.5 , 0.7 *and* 0.9

The last simulation analysis that we conducted assumes that genes are correlated, i.e. *ρ* > 0, since multiple studies have shown that the expression levels of genes from the same biological pathway are correlated [34, 35]. Since we have shown that the performances of Pathifier and our approach are very similar to each other and this observation is not strongly affected by the correlation between genes, we excluded the Pathifier from the comparison in this simulation analysis. Also, we set *p*_*G*_ to be 0.2. For different settings of *δ* and *ρ*, again, the optimal number of clusters is first identified to minimize the connectivity criterion. Then, this optimal number of clusters will be set to be the target number of clusters, and both hierarchical clustering and K-means clustering are applied to the three distances to identify the clusters. Since the correct number of clusters for all the simulated datasets is 3, we examined the percentage of simulated datasets that successfully identified 3 as the optimal number of clusters (success rate) based on the connectivity criterion (Figure 6). First, as can be seen in the figure, the pathway-based distance score achieves the highest success rate for almost all the examined values of *δ* and *ρ*. The Euclidean distance using all genes, again, has the lowest success rate, and the Euclidean distance using KEGG pathway annotated genes is between the other two distances. Second, the difference in the success rate is marginal when hierarchical clustering and K-means are used to calculate the connectivity criterion. Third, when the differences between groups (*δ*) are fixed, the success rate increases when the correlation between genes (*ρ*) increases. This increasing trend becomes weaker when the group difference is larger, especially for the pathway-based distance score. When *δ* = 1.5, the success rate of the pathway-based distance score has a decreasing trend, indicating that high correlation between genes makes it harder to identify the correct number of clusters when there is a big difference between the true classes. This could be due to the increase in the number of non-zero parameters to estimate in the covariance matrix for the Gaussian mixture model causing the model to be less efficient. Lastly, when the correlation between genes (*ρ*) is fixed, the success rate decreases when *δ* increases for the two Euclidean distances, especially when hierarchical clustering is used to calculate the connectivity criterion. For the pathway-based distance score, it is sensitive enough so that its success rate keeps increasing when the group difference (*δ*) increases for *ρ* < 0.9. Next, the purity criterion of the clustering results was examined by hierarchical clustering and K-means clustering using the three distances, which is shown in Fig. 7. These results show that, when the difference between groups (*δ*) is given, all three distances are less efficient when the correlation between genes (*ρ*) increases. This applies to both hierarchical clustering and K-means clustering. But when the correlation between genes (*ρ*) is given, the clustering results become increasingly accurate for all three distances when the differences between groups (*δ*) increase. In addition, the pathway-based distance score outperforms the other two distances for all given values of *δ* and *ρ*. Notably, this difference in performance is more significant for larger *ρ* when *δ* ≤ 0.9 and less significant for larger *ρ* when *δ* > 0.9. This differences in performance is also greater for larger *δ*, especially when *δ* > 1. Finally, the outer-performance of the pathway-based distance score over the other two distances is greater when hierarchical clustering is used, indicating the benefit of using K-means, especially when the number of clusters is correctly identified.

In summary, the comparison of the three distances shows that the pathway-based distance score achieves superior clustering results compared to the Euclidean distance, regardless of whether the correct set of genes are filtered out or not.

### Gene expression data in asthma patients

**Fig. 8:**
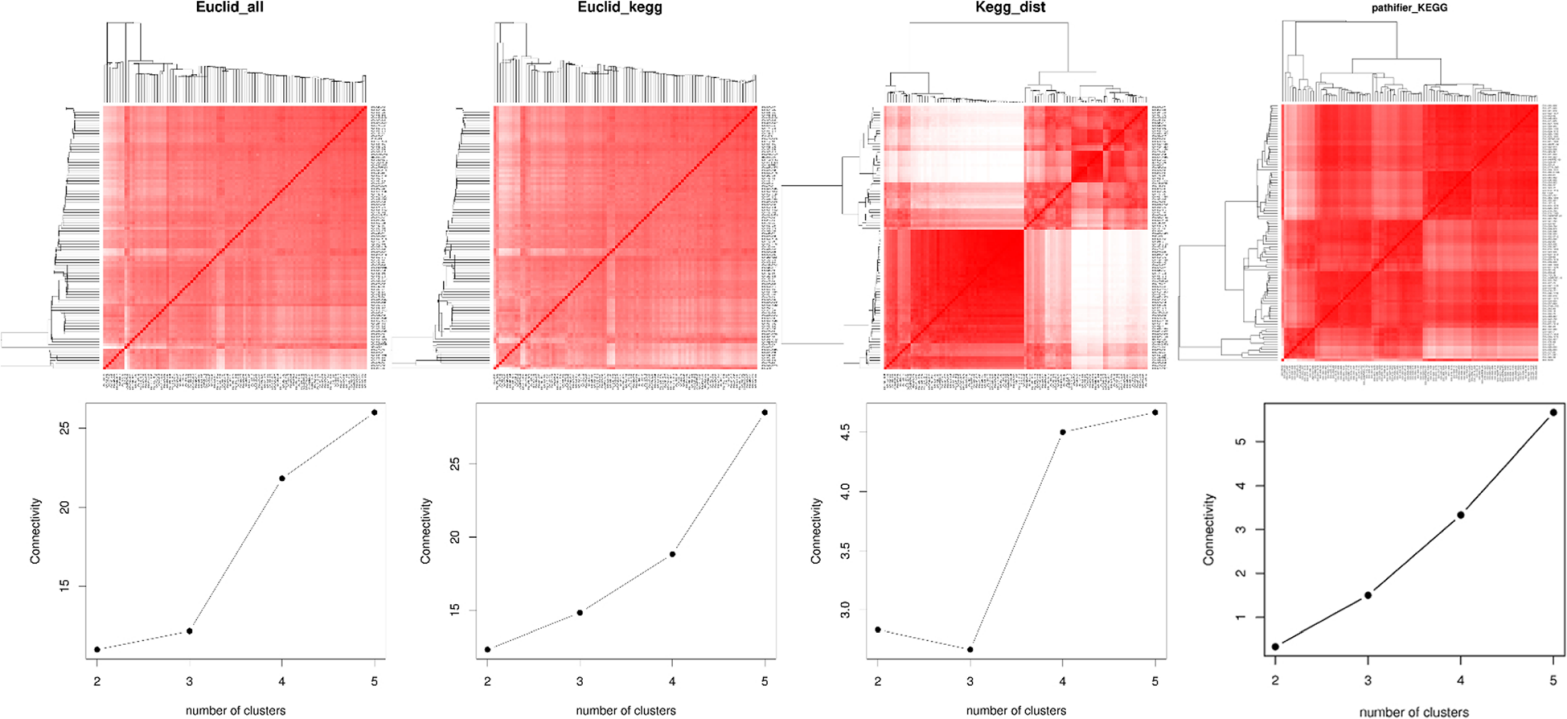
Distance matrices and connectivity criteria for the asthma gene expression data. Visualization and the connectivity criteria of the three distances for the YCAAD gene expression data in asthma patients. In the heatmaps on the top, rows and columns are both samples. Red represents smaller distance and white represents larger distance

To compare the three distances in real data, we applied them to the gene expression data measured in 100 asthma patients from the YCAAD cohort [7]. The data was quantile normalized and adjusted for batch effects using ComBat [36] and RIN numbers using linear regression. Then we calculated the three distances between the 100 patients using the adjusted gene expression data. The visualization and the connectivity criterion of the four distances are shown in Fig. 8. The heatmap shows that the pathway-based distance score indicates that there is a clear separation between samples, while there is no clear separation between samples using the other distances. The connectivity plot of the pathway-based distance shows that there should be 3 clusters and the other three distances achieve the smallest connectivity for 2 clusters.

Next, we clustered the 100 samples using K-means clustering coupled with the three distances, with *K* set to 3 for the pathway-based distance and 2 for the other three distances. Clinical and physiological features of the patients that are significantly associated (*P* < 0.05) with the clustering results are summarized in Table 2. As shown in the table, among all the clinical features, a history of hospitalizations and intubations are significant (*P* < 0.05) only by the pathway-based distance. These two features have already been validated in an independent cohort of children with asthma [7]. In addition, among all the physiological features, the significant features identified by the pathway-based distance score were also identified as significant by at least one of the other three distances, while the other two significant clinical features were only identified by one of the other three distances, indicating that they are likely to be false discoveries. In this asthma cohort, cytokine levels in the sputum and blood were also measured. When tested for association with the clustering results, 4 of them are significant only by the Euclidean distance with all genes, 2 of them are significant only by the Euclidean distance with KEGG pathways covered genes, 4 of them are significant only by Pathifier, and 6 of them are significant only by the pathway-based distance (data not shown). Since these cytokines are important proteins involved in inflammation and airway remodeling that have been shown to be important in the pathogenesis of asthma, we believe that the pathway-based distance was able to identify stronger signals of asthma heterogeneity from this data.

**Table 2:** Phenotypic and physiologic characteristics of the identified clusters

	Euclid_all	Euclid_KEGG	KEGG_dist	Pathifier_KEGG
Age at Visit (years)	0.65	0.37	0.32	0.28
Gender	0.02*	0.14	0.58	0.28
**History of Atopy - N (%)**	**0.89**	**0.2**	**0.02**	**0.62**
Age of Symptom Onset	0.55	0.25	0.17	0.62
Disease Duration (years)	0.98	0.9	0.67	0.38
**History of Hospitalization - N (%)**	**0.21**	**0.77**	**0.04**	**1.00**
**History of Intubations - N (%)**	**0.14**	**0.12**	**0.05**	**0.04**
OCS tapers in past year- N (%)	0.65	1.00	0.67	0.83
ACT Score	0.25	0.41	0.22	0.56
**FEV1- % of predicted value**				
**Pre β**_**2**_ **agonist use**	**0.04**	**0.02**	**0.02**	**0.04**
Post β_2_ agonist use	0.06	0.05*	0.06	0.06
FVC- % of predicted value				
**Pre β**_**2**_ **agonist use**	**0.04**	**0.02**	**0.04**	**0.03**
Post β_2_ agonist use	0.12	0.06	0.16	0.13
FEV1/FVC- % of predicted value				
Pre β_2_ agonist use	0.23	0.46	0.13	0.41
Post β_2_ agonist use	0.14	0.2	0.06	0.09
**BDR (%)**	**0.27**	**0.05**	**0.05**	**0.09**
**FENO (ppb)**	0.05*	0.54	0.27	0.40

The significance of the association between the phenotypic and physiologic features and the clustering results by K-means coupled with the four distances. *P* values were calculated using Kruskal-Wallis and Chi-square test for continuous and categorical variables, respectively. The false discovery rate for KEGG_dist clustering results associated clinical features estimated by the permutation-based method is 11% when nominal *p* value < 0.05. *P values that are significant (*P* < 0.05) only by one of the two Euclidean distances. Bold *p* values are significant by the pathway-based distance score

The gene expression data was adjusted for batch effects using ComBat, which may not completely eliminate the batch effects in the data. However, evaluation of the correlation between the clustering results with the batches showed that the clustering results were not significant correlated with the batches (data not shown). This indicates that the adjusted gene expression data was not dominated by the batch effect after the batch adjustment. Besides, as shown above, we validated our findings in an independent cohort that used the Illumina bead chips in a very different population of asthma patients. Combining these two facts, we concluded that the clustering results or the performance of our approach on the asthma gene expression data was not significantly affected by the batch effects. Nevertheless, we applied the methods to another real dataset with one single batch for demonstration, which can be found in the Supplemental Material (Additional file 1: Figure S10).

## Discussion

The pathway-based distance was calculated using genes included in pre-defined pathways. Thus, the biological significance of the identified clusters will rely heavily on the way that the pathways are defined. The KEGG pathways are mostly metabolic pathways that are ubiquitously involved in different complex diseases. However, if other types of pathways, for example, cancer-related pathways, are used to calculate the score, the biological difference that the score represents will be related to cancer-related pathways. Therefore, the best type of pathways to use will depend on the disease of interest. The cancer-related pathways may be limited but could provide stronger and more specific signals when applied to gene expression data in cancer patients. When choosing between different pathway databases, one may compare the percentage or the absolute number of pathways that identify more than 1 cluster from the data as this number may increase the resolution of the pathway-based distance. Clinical relevance of the identified clusters can be another way to help decide on which pathway database to use.

Second, the pre-defined pathways may be incomplete, contain errors and sometimes based on subjective judgments on the relevance of certain molecules with certain biological processes. When the pre-defined pathways are incomplete or the pathway database completely misses some of the important pathways related to the disease pathology, the molecular network changes associated with the disease may not always appear in the annotated pathways. In this case, the pathway-based clustering methods may be less effective than the gene-level clustering methods because of the lower signal level due to the gene filtering based on incomplete prior pathway annotation. Of course, when there are few disease pathology-associated genes but the differences between the different subgroups are high, the simulation showed that the pathway-based distance may still provide accurate clustering results. The subjective judgment on the pathway definitions will cause the clustering results to be different when different pathway databases are used, even though they contain the same type of pathways. These all indicate that one may need to refine the pre-defined pathways before calculating the pathway-based distance. This refinement includes both filtering genes and expanding the pathways to include more genes in a biologically meaningful way. In addition, applying the pathway-based clustering methods using multiple different pathway databases and examining the results for consistency are also recommended to obtain more robust and accurate clustering results.

Finally, the multivariate Gaussian mixture model is not the only way to identify the clusters using genes from each pathway. As we have shown in the high dimension simulation model, the approach can be significantly improved by adding an extra step of dimension reduction by downsampling the pathways into smaller pathways. In addition, other statistical models may be developed to better model the correlation between genes and the overlapping between different pathways. Alternative models are also needed to fit gene expression data measured by different techniques that do not follow Gaussian distribution, like the RNA sequencing data.

## Conclusions

We have developed a novel distance to represent the biological difference between samples using gene expression data. This distance has been compared to the traditional Euclidean distance with and without gene filtering using both simulated and real data as well as another pathway-based approach, Pathifier. The comparison in the simulated data sets showed that compared to the Euclidean distances with or without gene filtering, the pathway-based distance has better performance in both identifying the true number of clusters and assigning the samples to the correct classes that they truly belong to. This better performance is robust to the changes in the correlation between genes and the difference between different classes. When compared to Pathifier, the pathway-based distance showed better performance and robustness for pathways with a small number of genes. For pathways with a large number of genes, which causes the Gaussian mixture model to be less efficient due to the high dimension, we added an extra step of downsampling the pathways which showed significant improvement in the performance, especially in the accuracy of the clustering result. In the real dataset from asthma patients, compared to the two Euclidean distances, the pathway-based distance was the only distance that identified clinical features that are significantly different among the identified clusters. It was also the only approach that identified significant physiological features that were also significant by using at least one of the other distances. Finally, the pathway-based distance score identified the most number of inflammation and remodeling associated cytokines which were shown to be important biomarkers of different asthma pathogenesis. In the other real data set from non-small cell lung cancer patients, the pathway-based distance was able to achieve clustering accuracy comparable to the other methods, even though the small sample size (*n* = 11) caused the Gaussian mixture model to be less efficient for almost all the pathways. In summary, in both simulated data and real data, we have shown that the pathway-based distance provides accurate and robust clustering results which are more likely to be biologically meaningful.

## Additional file

Additional file 1: The pdf document that contains the application and comparison of the methods to the data from patients with non-small cell lung cancer, all supplementary notes and figures. **Figure S1** shows the median connectivity criterion of the pathway based distance score when the scoring matrix is treated as original data matrix instead of a distance matrix. **Figure S2** shows the standard deviation of the connectivity criteria of all the methods across the 100 simulated low dimension data. **Figure S3** shows the Dunn Index of all four methods as an alternative to the connectivity criterion using the low dimension simulation model. **Figure S4** shows the comparison across all the methods using low dimensional simulation model with 40% of genes in the chosen pathways to be perturbed. **Figure S5** shows the median and the standard deviation of the connectivity criterion of all four methods using the low dimension simulation model with a high background noise level (B = 3). **Figure S6** shows the Dunn Index of all four methods as an alternative to the connectivity criterion using the low dimension simulation model with a high background noise level (B = 3). **Figure S7** shows the performance comparison across the four methods using the low dimension simulation model with a high background noise (B = 3) and 40% of genes in the chosen pathways to be perturbed. **Figure S8** shows the performance comparison across the four methods using the high dimension simulation model with a lower percentage of genes in the chosen pathways to be perturbed (20% and 40%). **Figure S9** plots the purity of the clustering result by each pathway versus the number of genes in the corresponding pathway. **Figure S10** shows the visualization of the distance matrices calculated by the four methods and their corresponding connectivity criteria for the real gene expression data from patients with non-small cell lung cancer. (PDF 5361 kb)

## Abbreviations

BIC: Bayesian Inference Criterion
KEGG: Kyoto Encyclopedia of Genes and Genomes
MsigDB: Molecular Signatures Database.
