# Supplementary material for: A novel pathway-based distance score enhances assessment of disease heterogeneity in gene expression
Source: BMC Bioinformatics. 2017 Jun 20;18:309. doi: 10.1186/s12859-017-1727-4 (PMC5480187; doi:10.1186/s12859-017-1727-4)
Supplement: Additional file 1: — The pdf document that contains the application and comparison of the methods to the data from patients with non-small cell lung cancer, all supplementary notes and figures. Figure S1 shows the median connectivity criterion of the pathway based distance score when the scoring matrix is treated as original data matrix instead of a distance matrix. Figure S2 shows the standard deviation of the connectivity criteria of all the methods across the 100 simulated low dimension data. Figure S3 shows the Dunn Index of all four methods as an alternative to the connectivity criterion using the low dimension simulation model. Figure S4 shows the comparison across all the methods using low dimensional simulation model with 40% of genes in the chosen pathways to be perturbed. Figure S5 shows the median and the standard deviation of the connectivity criterion of all four methods using the low dimension simulation model with a high background noise level (B = 3). Figure S6 shows the Dunn Index of all four methods as an alternative to the connectivity criterion using the low dimension simulation model with a high background noise level (B = 3). Figure S7 shows the performance comparison across the four methods using the low dimension simulation model with a high background noise (B = 3) and 40% of genes in the chosen pathways to be perturbed. Figure S8 shows the performance comparison across the four methods using the high dimension simulation model with a lower percentage of genes in the chosen pathways to be perturbed (20% and 40%). Figure S9 plots the purity of the clustering result by each pathway versus the number of genes in the corresponding pathway. Figure S10 shows the visualization of the distance matrices calculated by the four methods and their corresponding connectivity criteria for the real gene expression data from patients with non-small cell lung cancer. (PDF 5361 kb) [file 12859_2017_1727_MOESM1_ESM.pdf]

A novel pathway-based distance score enhances assessment of disease heterogeneity in gene expression

Xiting Yan[1]*, Anqi Liang[2], Jose Gomez[1], Lauren Cohn[1] and Hongyu Zhao[1,2,3,4], Geoffrey L. Chupp[1]

Online Supplemental Materials

Application to Gene Expression Data in Non-small Cell Lung Cancer (NSCLC) Patients

The study in [1] measured gene expression profiles in 58 patients with non-small cell lung cancer (NSCLC) which has two major subtypes: adenocarcinoma (AC) and squamous cell carcinoma (SCC). There are 40 patients with AC and 18 patients with SCC. The 58 arrays were measured in different batches. To avoid the batch effect, we have chosen one single batch that has 5 AC patients and 6 SCC patients. The four methods were applied to these 11 samples to see if the 5 AC patients can be accurately separated from the 6 SCC patients. The heatmaps of the four distance matrices are shown in Figure S10, which shows very similar clustering results by all four methods. When applying K-means clustering to the four different distance matrices by setting K=2, euclidean distance using all genes, euclidean distance using KEGG genes and Pathifier using KEGG pathways all correctly assigned the 5 AC samples into the same cluster but all misclassified 2 SCC samples as AC samples. For our approach, it correctly assigned the 6 SCC samples into the same cluster but misclassified 3 AC samples as SCC samples. So our approach has a slightly higher but comparable misclassification rate. And the equal performance between the two Euclidean distances and the Pathifier indicates that the changes in the genomics and transcriptomics of these NSCLC patients are much larger than those in the chronic diseases, like asthma. The large changes drive the four methods to have similar results which are consistent with the results when δ is very high in the simulated data. One thing we want to point out is that in this dataset, we only have 11 samples which will make almost all the pathways have too many genes for the Gaussian mixture model which is a significant disadvantage for the pathway-based distance score. However, with this significant disadvantage, the pathway-based distance score is still able to achieve comparable performance as the other methods.

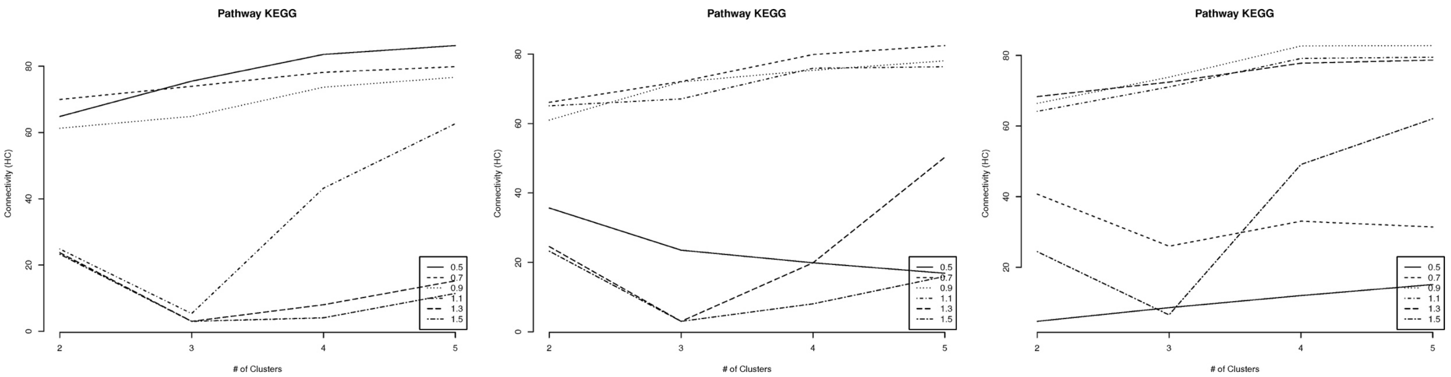

Figure S1: Median connectivity of the pathway based distance score for the low dimension simulation model when $\boldsymbol{B}$=1 and $\boldsymbol{p_G}$=0.8 (left panel), 0.6 (middle panel) and 0.4 (right panel). By comparing this plot to Figure 1, we do see significant improvement by treating the pathway based distance score as original data matrix instead of a distance matrix for clustering.

a

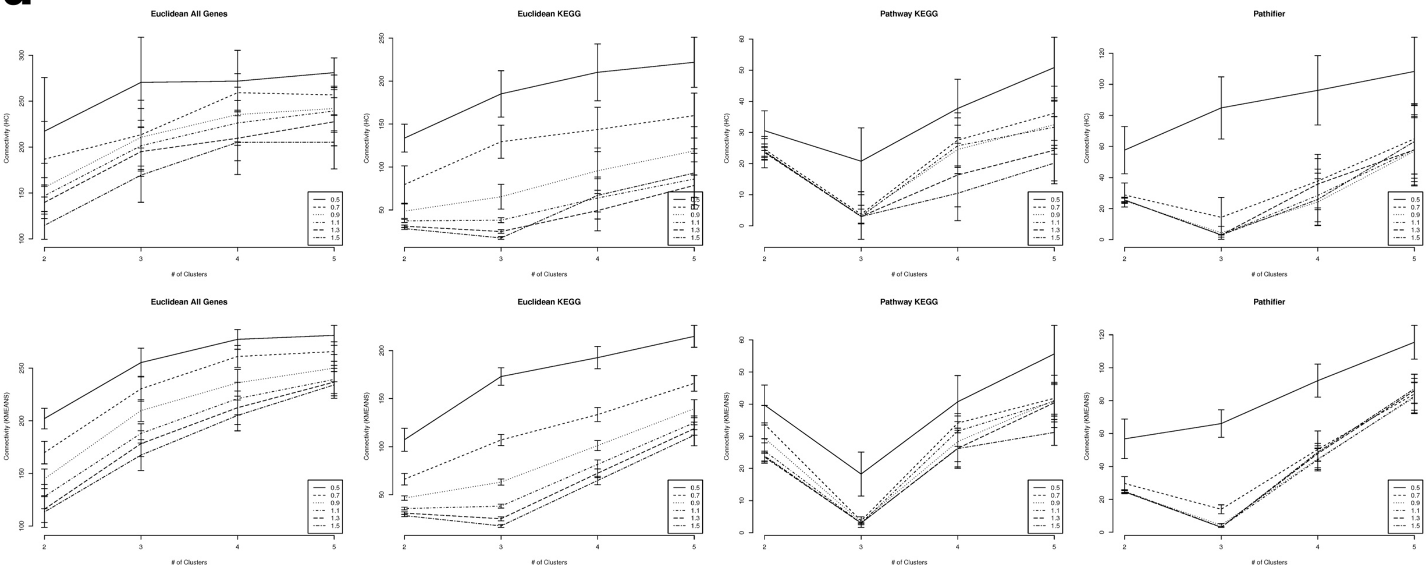

b

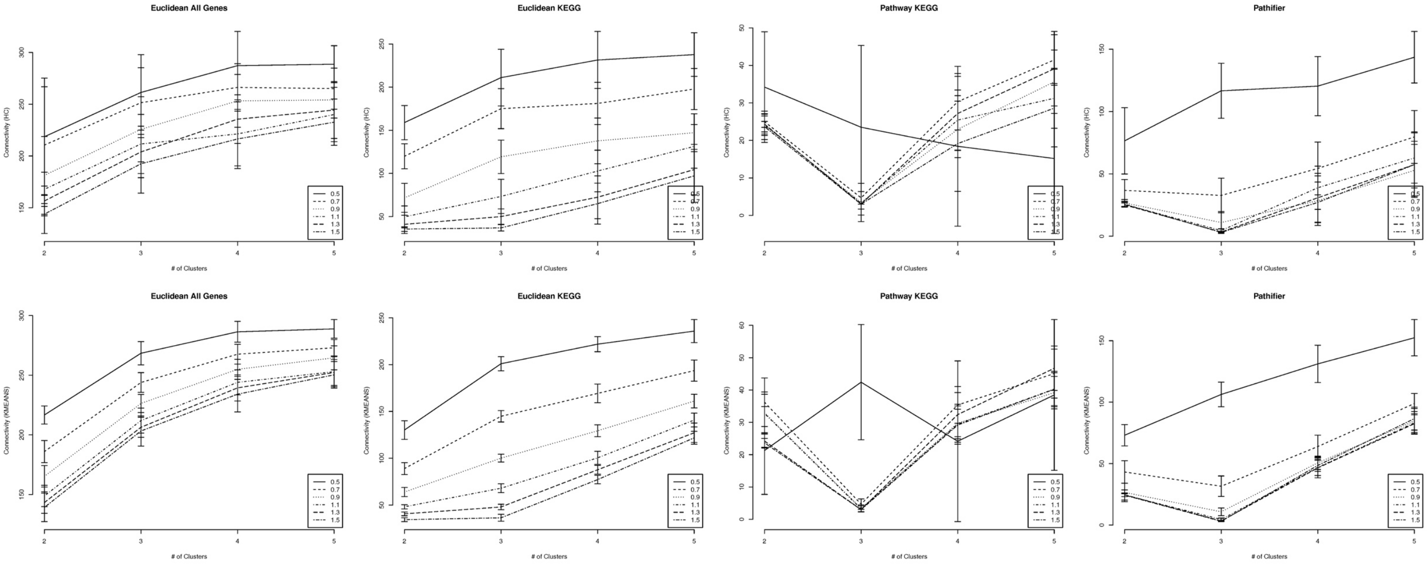

Figure S2: Median connectivity of the four distances with error bars on the low dimension simulation data when $\boldsymbol{B}$=1, $\boldsymbol{p_G}$=0.8 (panel **a**) and 0.6 (panel **b**).

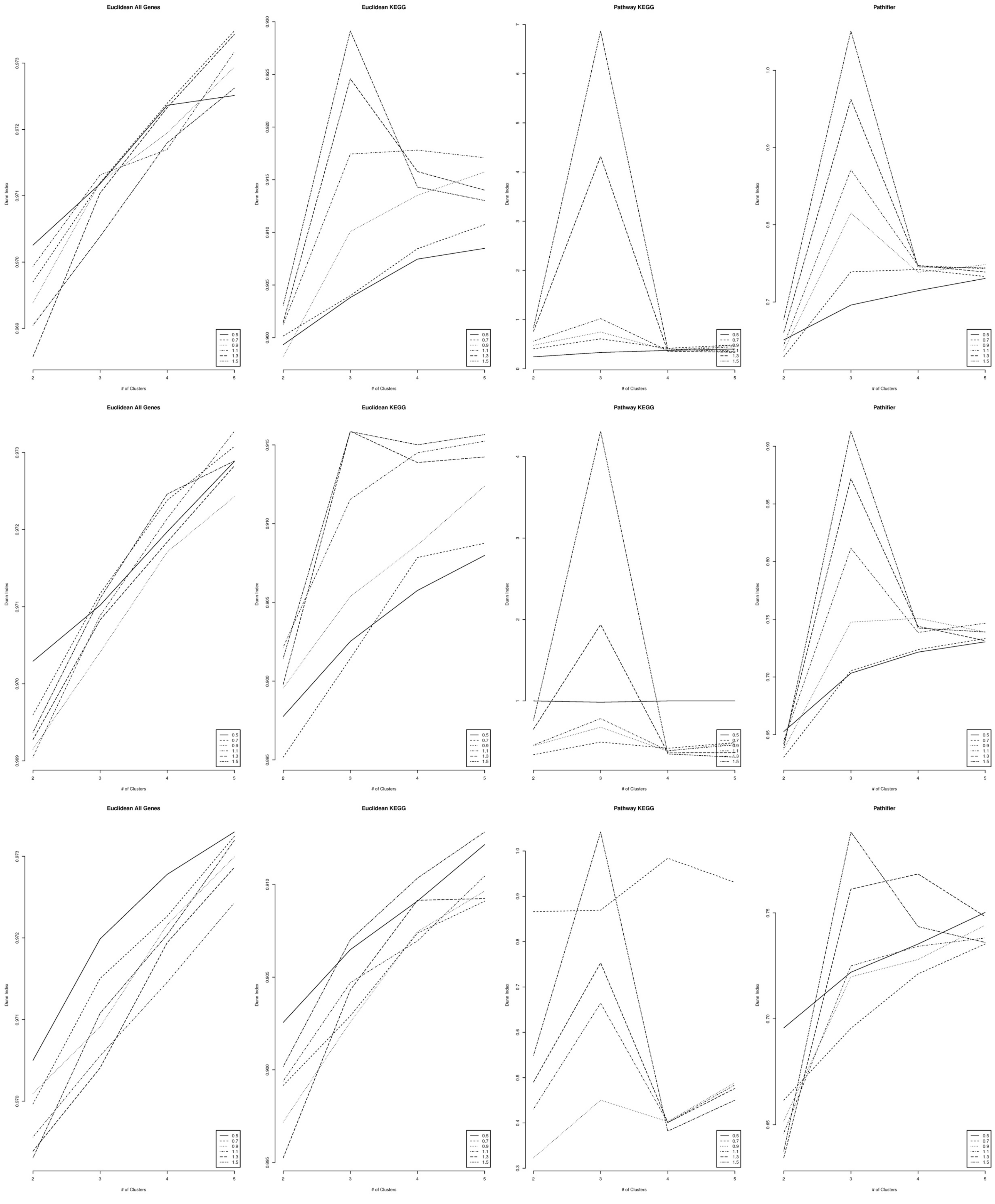

Figure S3: Dunn index for the low dimension simulation model when $\boldsymbol{B}$=1, $\boldsymbol{p_G}$=0.4 (bottom row), 0.6 (middle row) and 0.8 (top row).

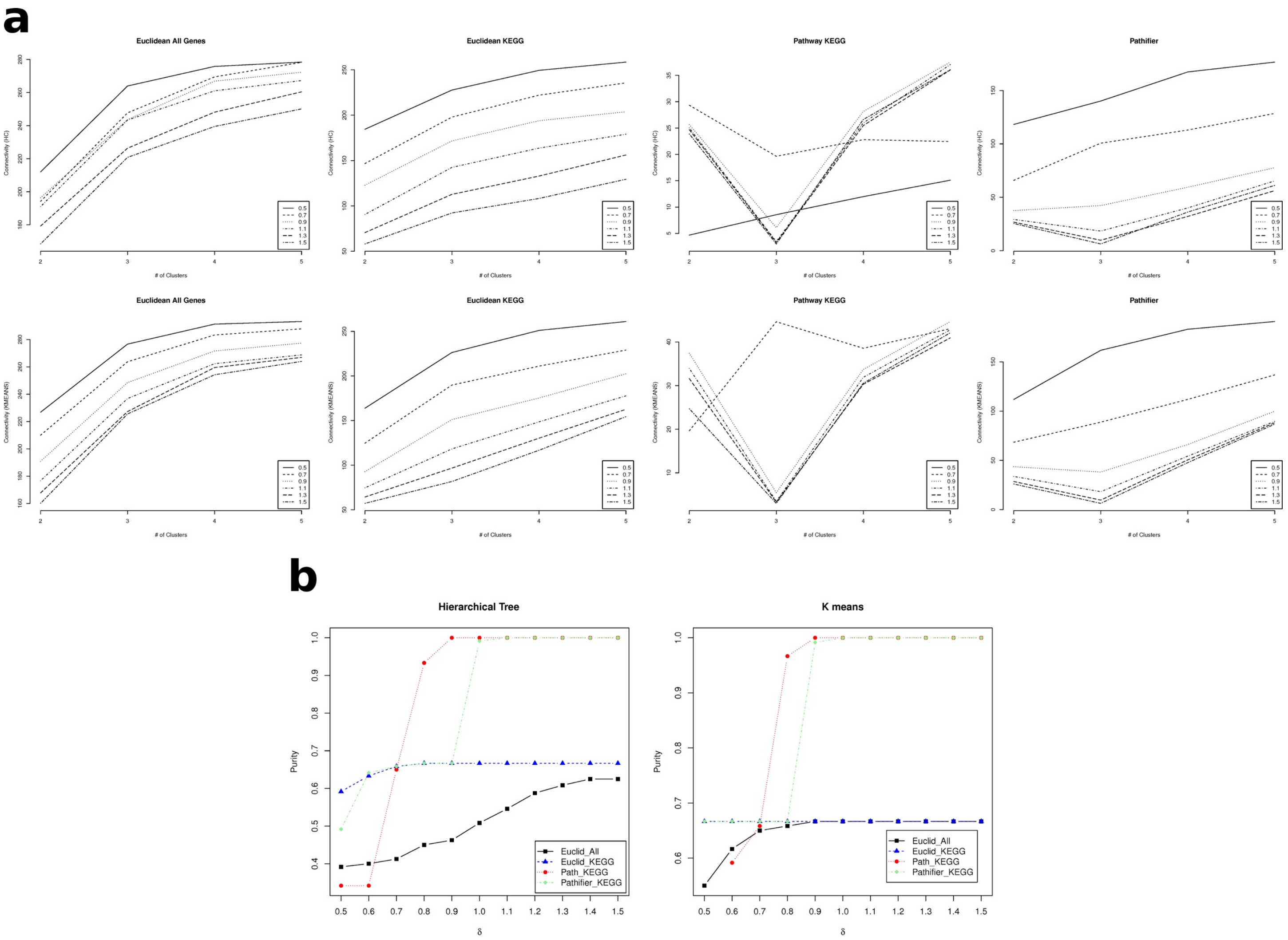

Figure S4: Median connectivity (panel **a**) and median purity (panel **b**) for different numbers of clusters when $\boldsymbol{B = 1}$ and $\boldsymbol{p_G = 0.4}$ for the low dimension simulation model.

a

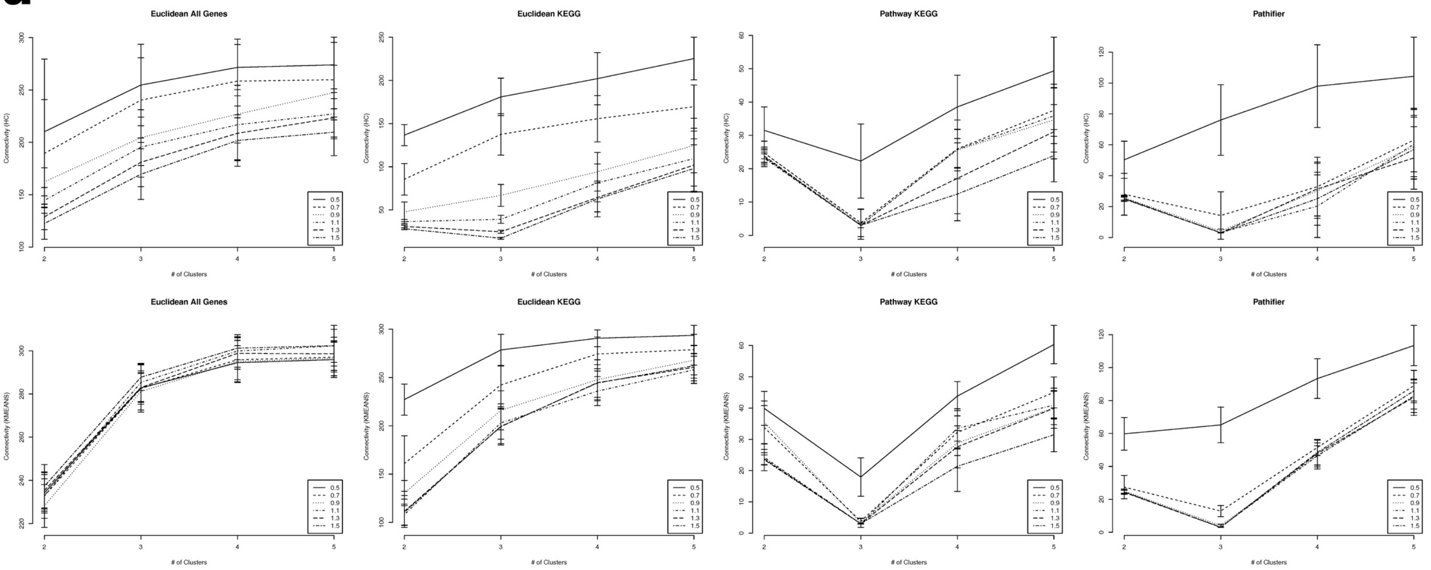

b

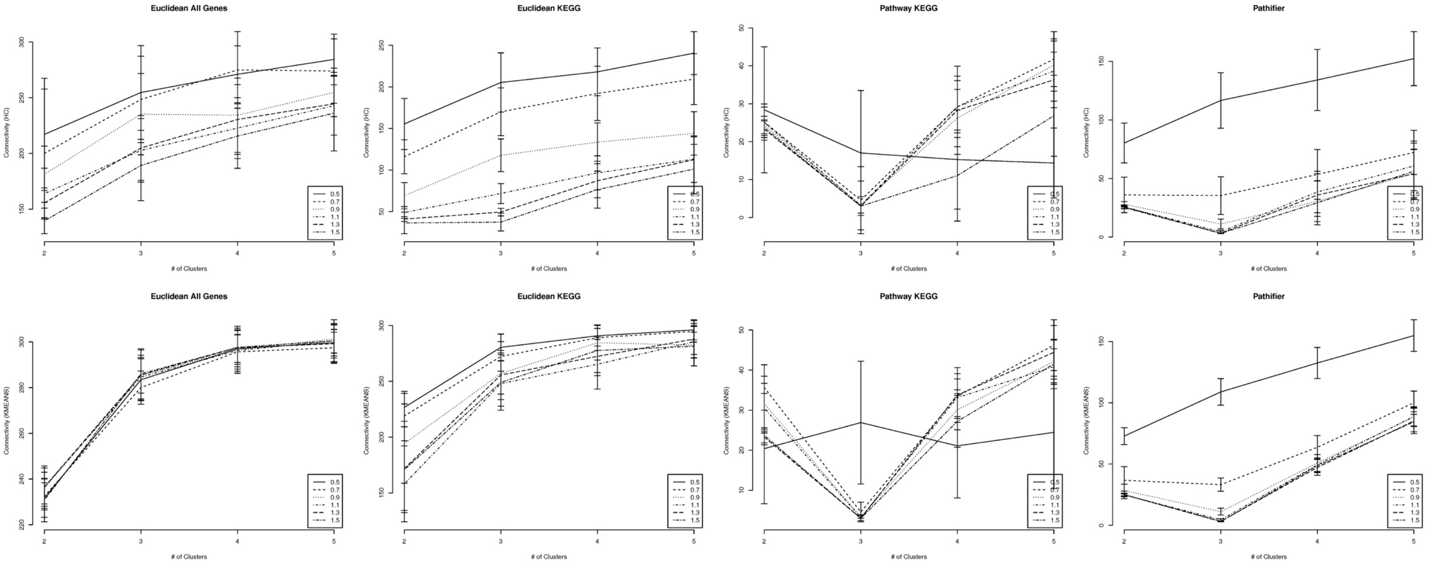

Figure S5: Median connectivity of the four distances with error bars on the low dimension simulation data when B=3, p_G=0.8 (panel **a**) and 0.6 (panel **b**).

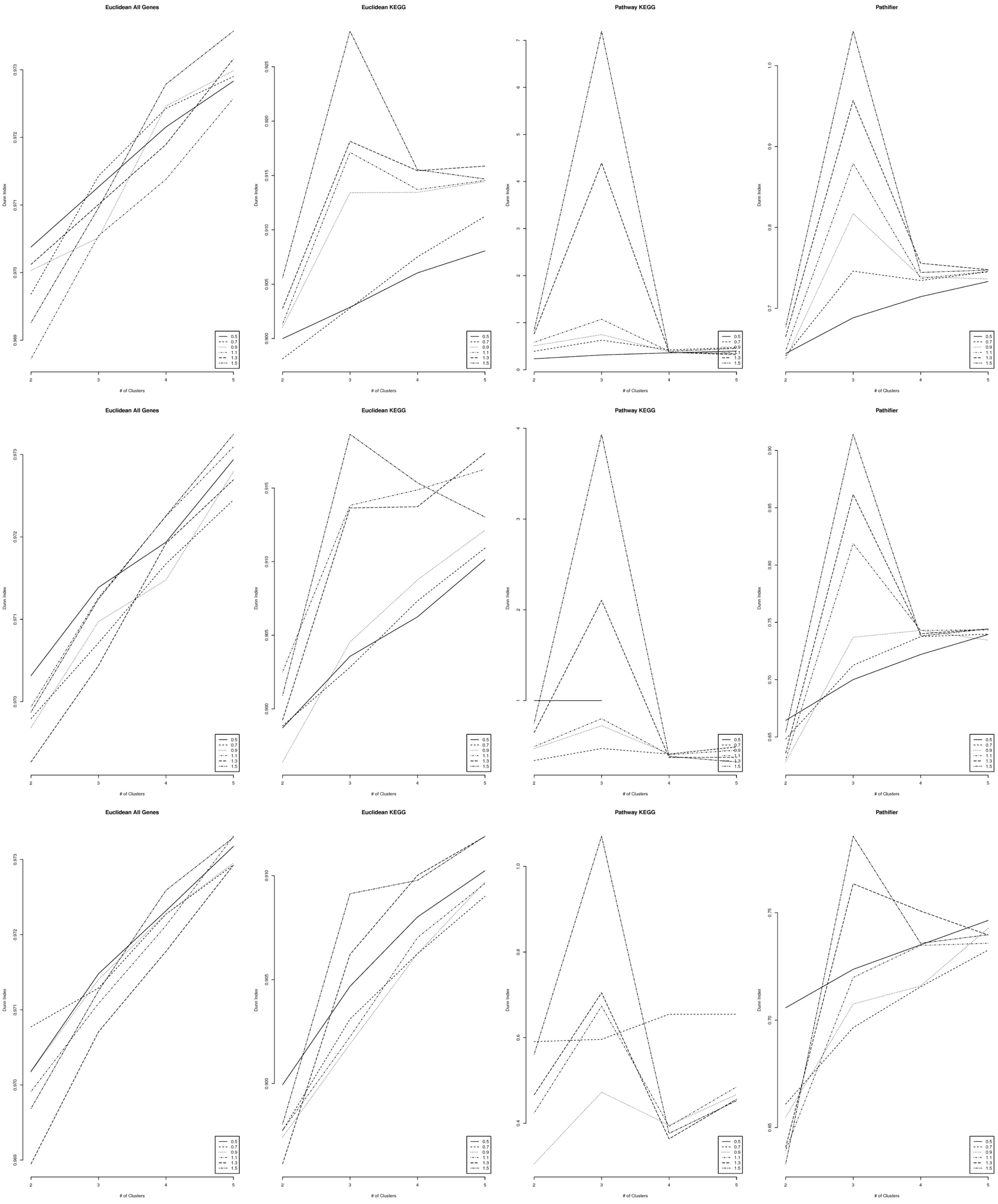

Figure S6: Dunn index for the low dimension simulation model when B=3, $\boldsymbol{p_G}$=0.4 (bottom row), 0.6 (middle row) and 0.8 (top row).

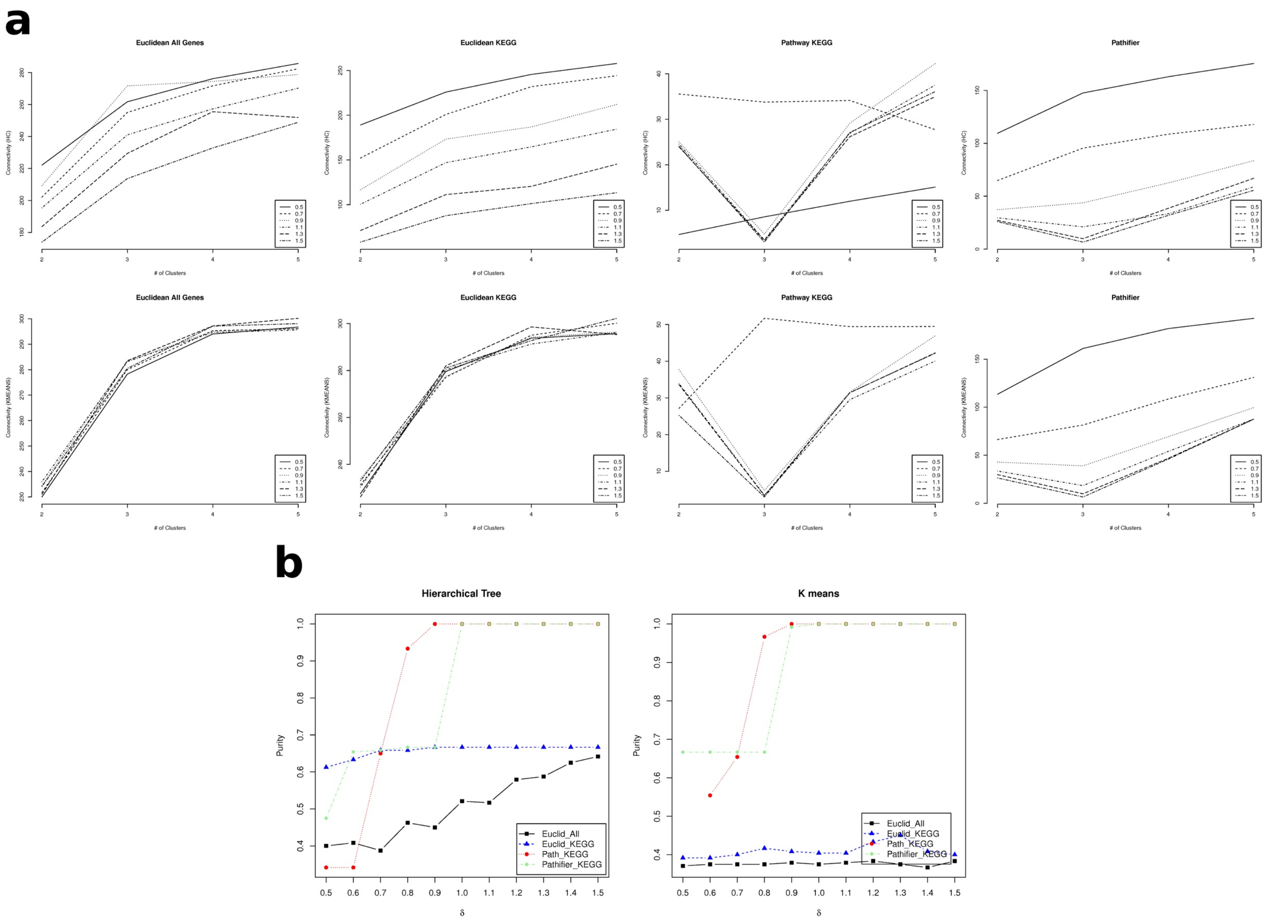

Figure S7: Median connectivity (panel **a**) and median purity (panel **b**) for different numbers of clusters when $\boldsymbol{B = 3}$ and $\boldsymbol{p_G = 0.4}$ for the low dimension simulation model.

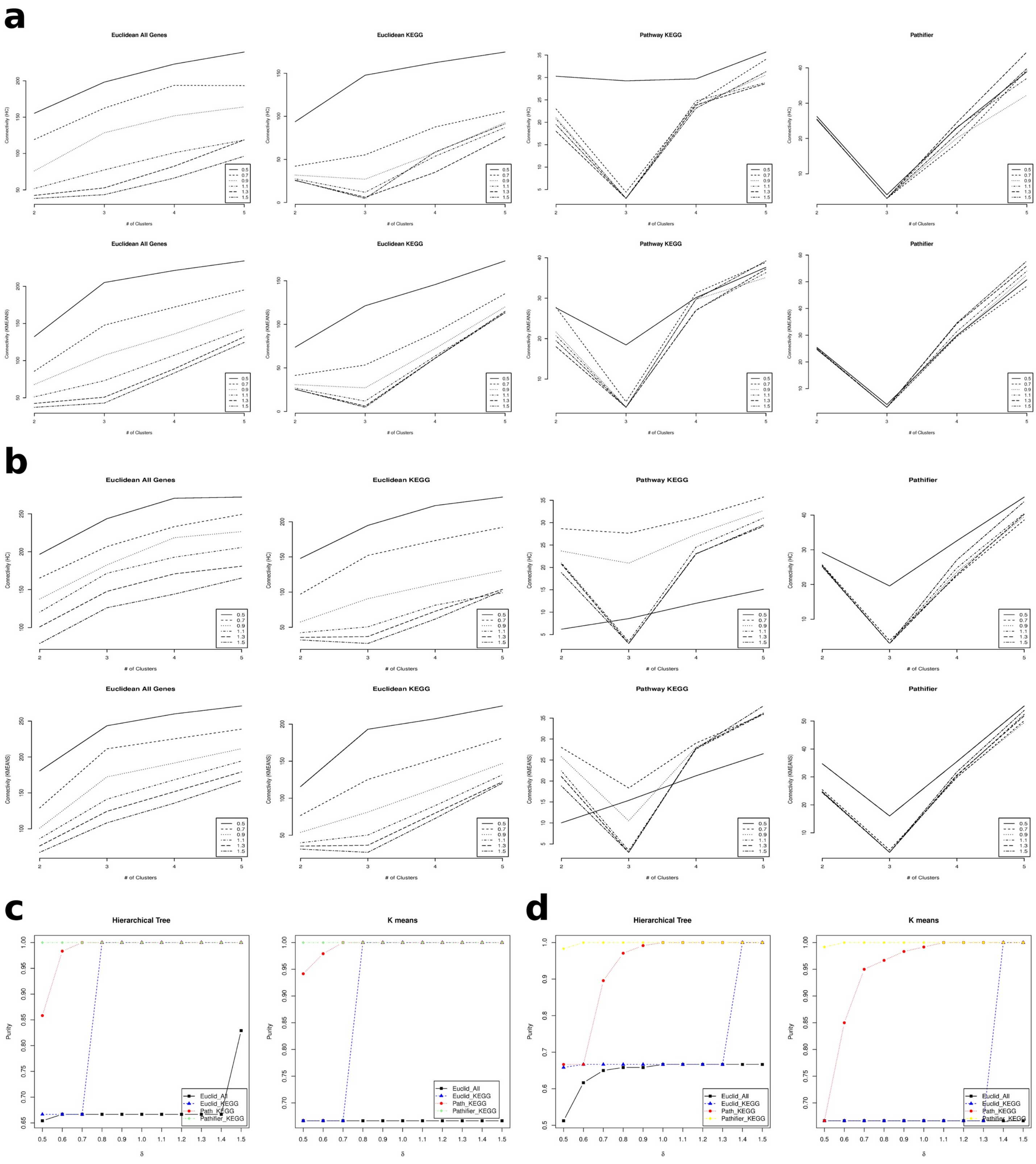

Figure S8: Connectivity for p_G=0.4 (panel **a**), 0.2 (panel **b**) and purity for p_G=0.4(panel **c**), 0.2 (panel **d**) for different numbers of clusters when B=1 in the high dimension simulation model.

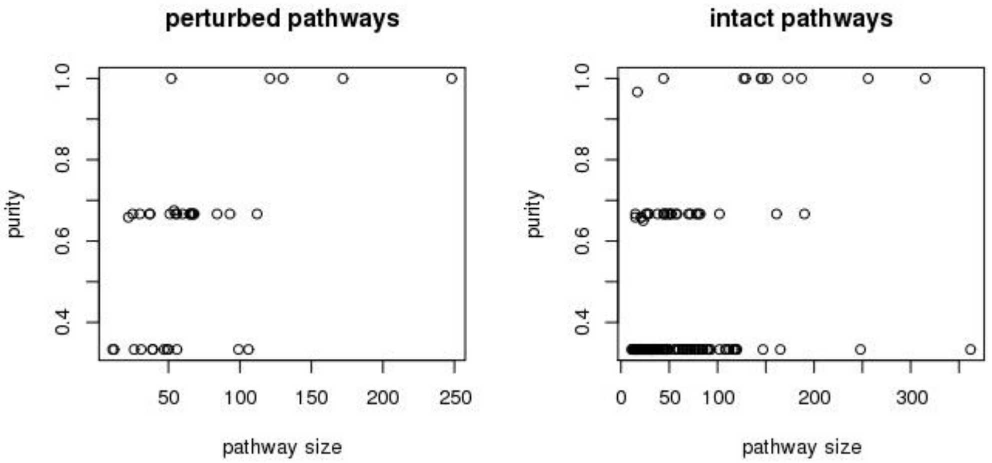

Figure S9: Purity of the clustering results by each pathway against the number of genes (pathway size). **Left panel** shows the data for the randomly chosen pathways to be perturbed (perturbed pathways) and **right panel** is for the other pathways (intact pathways). Because of the overlap between different KEGG pathways, the inatct pathways also contain signal genes which cause them to be able to achieve high purity even though they were not chosen to be perturbed.

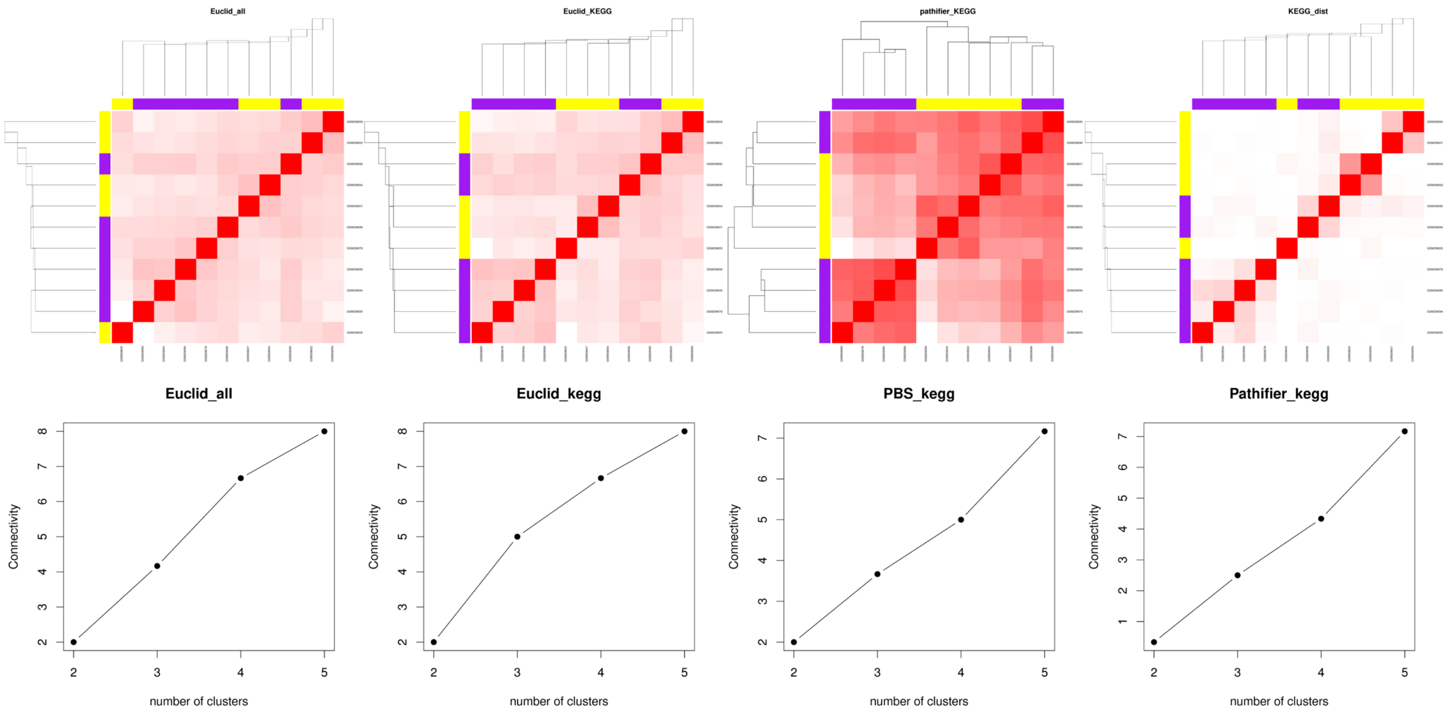

Figure S10: The distance matrices and connectivity criteria for the NSCLC gene expression data. Visualization and the connectivity criteria of the four distances for the gene expression data in non-small cell lung cancer patients. In the heatmaps on the top, rows and columns are both samples. The color bars at the edge of the heatmaps represent the true subclass (purple for AC and yellow for SCC) of the samples. Red represents smaller distance and white represents larger distance.

REFERENCES

1. Kuner R, Muley T, Meister M, Ruschhaupt M, Buness A, Xu EC, Schnabel P, Warth A, Poustka A, Sultmann H *et al*: **Global gene expression analysis reveals specific patterns of cell junctions in non-small cell lung cancer subtypes**. *Lung cancer* 2009, **63**(1):32-38.
